# ﻿Male-based key to the subfamilies and genera of Malagasy ants (Hymenoptera, Formicidae)

**DOI:** 10.3897/zookeys.1213.120531

**Published:** 2024-09-27

**Authors:** Manoa M. Ramamonjisoa, Nicole Rasoamanana, Brian L. Fisher

**Affiliations:** 1 Madagascar Biodiversity Center, BP 6257, Parc Botanique et Zoologique de Tsimbazaza, Antananarivo, Madagascar Madagascar Biodiversity Center Antananarivo Madagascar; 2 Entomology, California Academy of Sciences, 55 Music Concourse Drive, San Francisco, CA 94118, USA California Academy of Sciences San Francisco United States of America

**Keywords:** Formicidae, identification, Malagasy region, male ants, morphology

## Abstract

The males of the family Formicidae of the Malagasy region, including the islands of the southwest Indian Ocean (Madagascar, Mauritius, Reunion, Comoros, and Seychelles) are reviewed. A male-based synopsis of each subfamily and genera are provided. A richly illustrated male-based key to the eight subfamilies and 72 genera for which males are known are provided. The key is specific to the ant genera and species of the Malagasy region. Terminologies for morphology and wing cells are also reviewed. The keys are a product of three decades of collecting across the region. Despite efforts to collect males for all genera, males from five genera (*Brachyponera*, *Chrysapace*, *Dicroaspis*, *Linepithema*, *Ochetellus*) were included in the keys based on males from species collected outside the region, and males from one genus (*Parvaponera*) are unknown globally and not included in the key.

## ﻿Introduction

Most identification tools for ants are based on the worker female caste and neglect the male caste. Identifying males is important to understanding the life history, phenology, and reproductive biology of ants. In addition, some collecting methods like Malaise and light traps preferentially trap males and, without tools for their identification, limit the insights these methods can provide into ant community diversity and structure through time and space.

In the Malagasy region (Madagascar, Mauritius, Reunion, Comoros, and Seychelles), there has been a pioneering effort to develop the taxonomic tools to identify male ants to genus: Ponerinae (Yoshimura and Fisher 2007), Amblyoponinae (Yoshimura and Fisher 2012), Dolichoderinae (Yoshimura and Fisher 2011), Proceratiinae (Yoshimura and Fisher 2009), and Myrmicinae tribes (Ramamonjisoa et al. 2023). This body of work has greatly enriched our understanding of the diversity of ants in the region. Borowiec (2016) also provided an identification key for male Dorylinae from the African and Malagasy regions. Here, we update this previous work, provide additional characters and present keys to all genera, including the Myrmicinae for which males are known. The newly proposed key uses a combination of morphological characters to create a navigational tool to identify the diversity of ant genera in the Malagasy region. The effectiveness of the key is enhanced by the integration of photographic illustrations, which provide a visual portal to the subtle intricacies that distinguish each genera. This study aims to increase the accessibility, accuracy, and applicability of ant genera identification in the Malagasy region.

## ﻿Materials and methods

Morphological observations were carried out using Leica stereoscopic microscopes (MZ9.5). Digital color montage images were created using a JVC KY-F75 digital camera and Syncroscopy Auto-Montage software (v. 5.0), or a Leica DFC 425 camera in combination with the Leica Application Suite software (v. 3.8). These images are available online through AntWeb.org (2022) and are accessible using the unique specimen identifier code.

Terminology for general morphology follows Bolton (1994) and Boudinot (2013, 2015). The terminology for forewing venation follows Yoshimura and Fisher (2007) and for hindwing venation follows Yoshimura and Fisher (2011). When referring to the presence or absence of veins in the descriptions, a vein is considered present regardless of whether it is tubular, nebulous, or spectral (Mason 1986).

### ﻿Subfamilies and genera of the Malagasy Region

**Figure 1.**
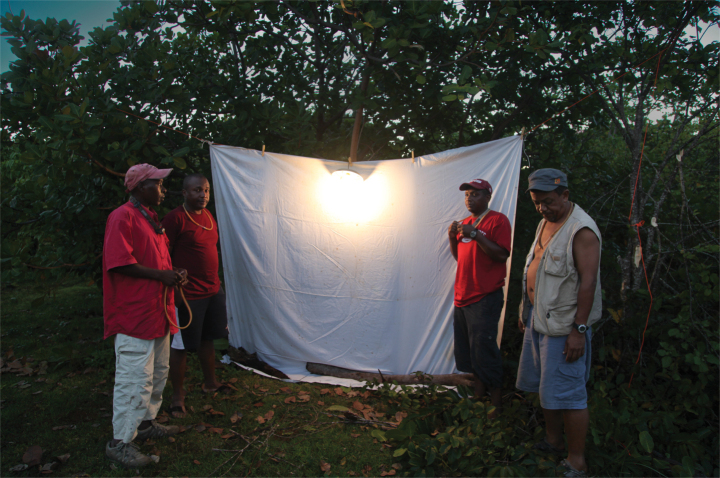
Black light. Photographer Brian Fisher.

**Figure 2.**
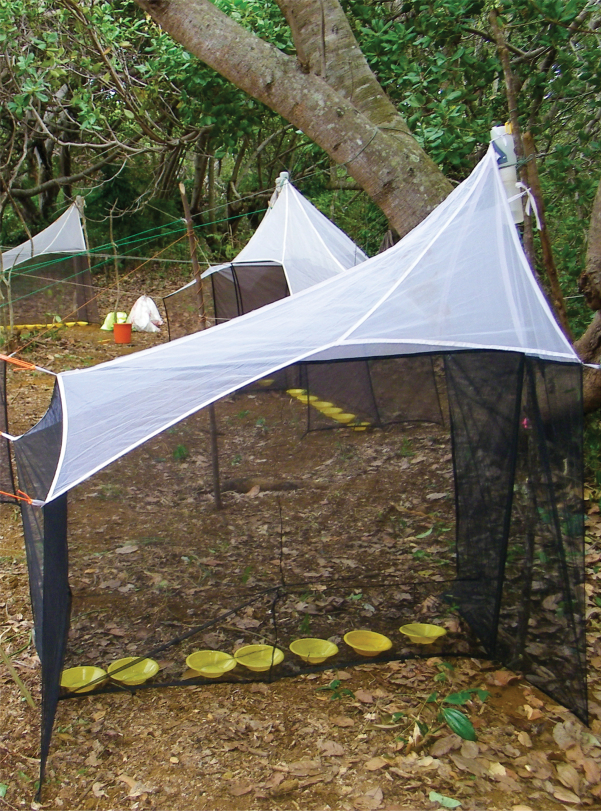
Yellow pan and Malaise trap. Photographer Brian Fisher.

The specimens used in this study are the product of a long-term effort to document the diversity of ants in the Malagasy region (Fisher 2005; Fisher and Peeters 2019). Males were collected by hand as part of colony series but also in light and Malaise traps. Despite these efforts, representative males have not been collected for all genera in the Malagasy region. Five genera (*Brachyponera*, *Chrysapace*, *Dicroaspis*, *Linepithema*, *Ochetellus*) have males known only from outside the Malagasy region. Males of *Brachyponera* (known from Mauritius), *Dicroapsis* (from Anjouan), *Linepithema* (from Reunion) and *Ochetellus* (from Reunion) are most likely absent because of the limited effort spent targeting these taxa on those islands. It is surprising that males have never been collected in the region for *Chrysapace*, a large Doryline present in northern Madagascar, despite the numerous Malaise and light traps placed throughout the range of the genus. Even more puzzling is the complete global absence of males of *Parvaponera*, as *Parvaponera* queens are regularly collected at black lights (Fig. 1). For a period of seven years, the Madagascar ant team directed efforts to collect males and locate colonies at sites where *Parvaponera* queens were present at lights. At one site, Nosy Faly in NW Madagascar, we located the first ground nest including workers for the genus in Madagascar. We set a series of yellow pan traps and Malaise traps during the period queens were present at black lights (Fig. 2), but no males were collected. Males of the genus remain unknown in Madagascar and globally. *Parvaponera* is the only genus in the Malagasy region absent from the key.

## ﻿Synoptic list of 73 ant genera of the Malagasy Region

For genera absent from Madagascar, the distribution is indicated in parentheses.

* Males unknown for the genus within the Malagasy region but included in keys based on males from outside the region.

+ Males unknown for genus globally and not included in key.

**AMBLYOPONINAE Forel, 1893**

1. *Adetomyrma* Ward, 1994

2. *Mystrium* Roger, 1862

3. *Prionopelta* Mayr, 1866

4. *Stigmatomma* Roger, 1859

5. *Xymmer* Santschi, 1914

**DOLICHODERINAE Forel, 1878**

1. *Aptinoma* Fisher, 2009

2. Linepithema* Mayr, 1866 (Reunion)

3. Ochetellus* Shattuck, 1992 (Mauritius, Reunion)

4. *Ravavy* Fisher, 2009

5. *Tapinoma* Foerster, 1850

6. *Technomyrmex* Mayr, 1872

**DORYLINAE Leach, 1815**

1. *Eburopone* Borowiec, 2016

2. Chrysapace* Crawley, 1924

3. *Lioponera* Mayr, 1879

4. *Lividopone* Bolton & Fisher, 2016

5. *Ooceraea* Roger, 1862

6. *Parasyscia* Emery, 1882

7. *Simopone* Forel, 1891

8. *Tanipone* Bolton & Fisher, 2012

**FORMICINAE Latreille, 1809**

1. *Anoplolepis* Santschi, 1914 (Seychelles)

2. *Brachymyrmex* Mayr, 1868

3. *Camponotus* Mayr, 1861

4. *Lepisiota* Santschi, 1926

5. *Nylanderia* Emery, 1906

6. *Paraparatrechina* Donithorpe, 1947

7. *Paratrechina* Motschoulsky, 1863

8. *Plagiolepis* Mayr, 1861

9. *Tapinolepis* Emery, 1925

**MYRMICINAE Lepeletier de Saint-Fargeau, 1835**

1. *Adelomyrmex* Emery, 1897 (Seychelles)

2. *Aphaenogaster* Mayr, 1853

3. *Calyptomyrmex* Emery, 1887 (Comoros)

4. *Cardiocondyla* Emery, 1869

5. *Carebara* Westwood, 1840

6. *Cataulacus* Smith, 1853

7. *Crematogaster* Lund, 1831

8. *Cyphomyrmex* Mayr, 1862 (Reunion)

9. Dicroaspis* Emery, 1908 (Comoros)

10. *Erromyrma* Bolton & Fisher, 2016

11. *Eurhopalothrix* Brown & Kempf, 1961 (Comoros)

12. *Eutetramorium* Emery, 1899

13. *Malagidris* Bolton & Fisher, 2014

14. *Melissotarsus* Emery, 1877

15. *Meranoplus* Smith, 1853

16. *Metapone* Forel, 1911

17. *Monomorium* Mayr, 1855

18. *Nesomyrmex* Wheeler, 1910

19. *Pheidole* Westwood, 1839

20. *Pilotrochus* Brown, 1978

21. *Pristomyrmex* Mayr, 1866 (Mauritius)

22. *Royidris* Bolton & Fisher, 2014

23. *Solenopsis* Westwood, 1840

24. *Strumigenys* Smith, 1860

25. *Syllophopsis* Santschi, 1915

26. *Terataner* Emery, 1912

27. *Tetramorium* Mayr, 1855

28. *Trichomyrmex* Mayr, 1865

29. *Vitsika* Bolton & Fisher, 2014

30. *Vollenhovia* Mayr, 1865 (Seychelles)

**PONERINAE Lepeletier de Saint-Fargeau, 1835**

*1. Anochetus* Mayr, 1861

2. *Bothroponera* Mayr, 1862

3. Brachyponera* Emery, 1900 (Mauritius)

4. *Euponera* Forel, 1891

5. *Hypoponera* Santschi, 1938

6. *Leptogenys* Roger, 1861

7. *Mesoponera* Emery, 1900

8. *Odontomachus* Latreille, 1804

9. *Parvaponera*+ Schmidt & Shattuck, 2014

10. *Platythyrea* Roger, 1863

11. *Ponera* Latreille, 1804

**PROCERATIINAE Emery, 1895**

1. *Discothyrea* Roger, 1863

2. *Probolomyrmex* Mayr, 1901

3. *Proceratium* Roger, 1863

**PSEUDOMYRMICINAE Smith, 1952**

1. *Tetraponera* Smith, 1852

### ﻿Male-based key to the subfamilies of the Malagasy Region

**Table:** 

1	Two distinct, long, narrow spines present on the posterior portion of abdominal sternum IX (Fig. 3A) or, if absent, then mandibles extremely elongated, distinctly longer than head, and volsella massive, claw-shaped, directed dorsally. Pygostyles absent	**Dorylinae**
–	Two distinct, long, narrow spines absent on the posterior portion of abdominal sternum IX (Fig. 3B). Mandibles not elongated or distinctly shorter than head. Volsella moderate, not directed dorsally. Pygostyles present or absent	**2**
2	Abdominal segment II nearly as large as or **longer than** segment III (postpetiole) in lateral view (Fig. 4A)	**3**
–	Abdominal segment II much **shorter than** segment III in lateral view (Fig. 4B)	**4**
3	Ventral apex of meso- and metatibia, when viewed from the front with the femur at right angle to the body, with two spurs consisting of a large pectinate spur and a small simple spur (Fig. 5A)	**Pseudomyrmicinae**
–	Ventral apex of metatibia, when viewed from the front with the femur at right angle to the body, with single simple spur or absent (Fig. 5B)	**Myrmicinae**
4	Metatibia with one or two ventroapical spurs; if only one spur present then cinctus distinct and deep between abdominal segment III and abdominal segment IV (Fig. 6A)	**5**
–	Metatibia always with single ventroapical spur, cinctus always indistinct between abdominal segment III and abdominal segment IV (Fig. 6B)	**7**
5	Anal region of hind wing vestigial (Fig. 7A) and with the mesosoma in lateral view, oblique mesopleural furrow reaching pronotum close to pronotal posteroventral margin (Fig. 7C)	**Proceratiinae**
–	Anal region of hind wing well developed (Fig. 7B); if vestigial, oblique mesopleural furrow always reaching pronotum far from pronotal posteroventral margin, or oblique mesopleural furrow absent (Fig. 7D)	**6**
6	Abdominal segment II broadly and dorsally attached to abdominal segment III; mandible long, falcate, curved inward and closed (Fig. 8A)	**Amblyoponinae**
–	Abdominal segment II narrowly and ventrally attached to abdominal segment III; mandible short, linear, subtriangular to triangular (Fig. 8B)	**Ponerinae**
7	With head in full-face view, scape short, not reaching posterior margin of head (Fig. 9A)	**Dolichoderinae**
–	With head in full-face view, scape long, distinctly **exceeding posterior** margin of head (Fig. 9B)	**Formicinae**

**Figure 3.**
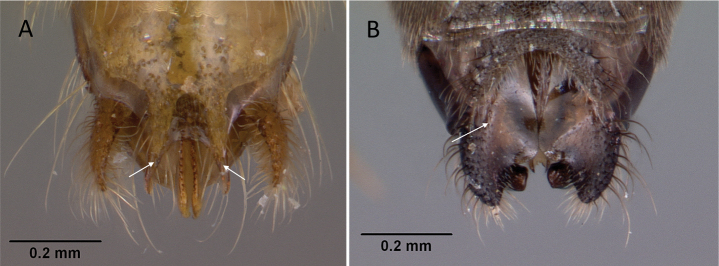
Portion of abdominal sternum IX **A***Lioponera* sp. (CASENT0001042) **B***Technomyrmex* mg08 (CASENT0049527). Photographer Masashi Yoshimura.

**Figure 4.**
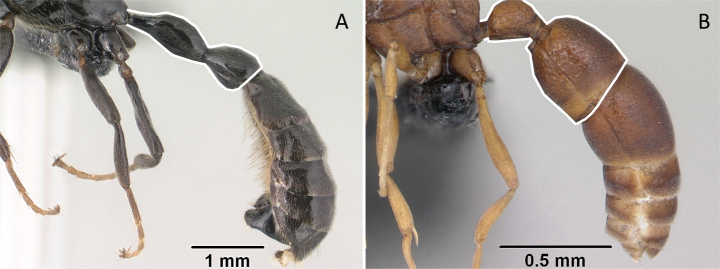
Abdominal segment II and III in lateral view **A***Tetraponeralongula* (CASENT0138661) **B***Probolomyrmexcurculiformis* (CASENT0050214). Photographers Dimby Raharinjanahary (**A**), April Nobile (**B**).

**Figure 5.**
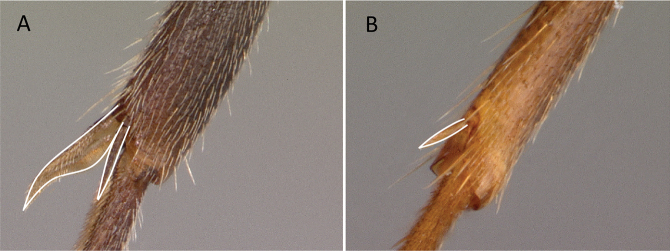
Metatibial spur **A***Tetraponera* psw094 (CASENT0053316) **B***Aphaenogasterswammerdami* (CASENT0000990). Photographers April Nobile (**A**), Masashi Yoshimura (**B**).

**Figure 6.**
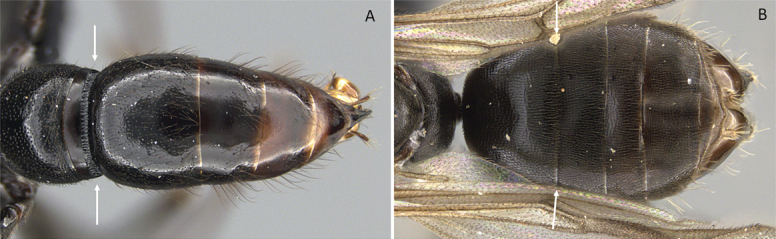
Gaster in dorsal view, cinctus at abdominal segment IV level **A***Euponerasikorae* (CASENT0065480) **B***Technomyrmexalbipes* (CASENT0055727). Photographer Michele Esposito.

**Figure 7.**
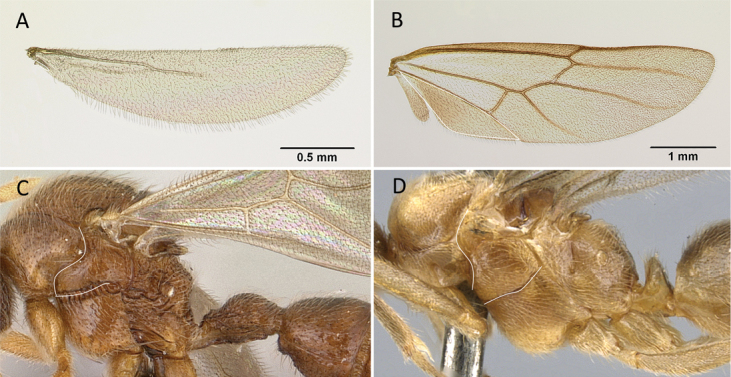
Hindwings of male ants **A***Discothyrea* mgm01 (CASENT0083649) **B***Odontomachuscoquereli* (CASENT0049797). Mesosoma in lateral view, showing oblique mesopleural furrow **C***Proceratium* dr01 (CASENT0145100) **D***Acropygagoeldii* (CASENT0903184). Photographers Erin Prado (**A**, **B**), Dimby Raharinjanahary (**C**), Ziv Lieberman (**D**).

**Figure 8.**
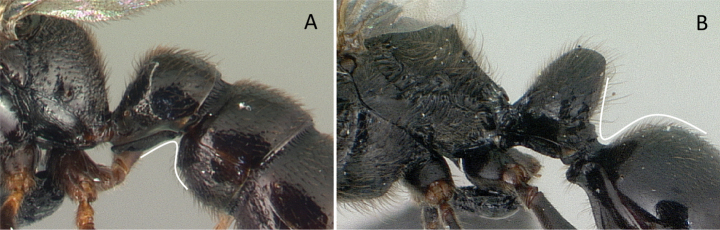
Attachment of abdominal segment II to abdominal segment III **A***Stigmatomma* mgm04 (CASENT0063981) **B***Bothroponeraperroti* (CASENT0135783). Photographers Erin Prado (**A**), Dimby Raharinjanahary (**B**).

**Figure 9.**
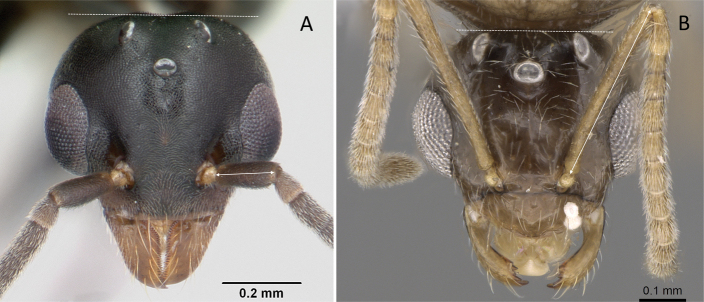
Head in full-face view showing the comparison of scape length **A***Technomyrmexalbipes* (CASENT0055727) **B***Lepisiotacanescens* (CASENT0906461). Photographers April Nobile (**A**), Cerise Chen (**B**).

#### ﻿AMBLYOPONINAE Forel, 1893

Diagnosis of male ants of the subfamily Amblyoponinae in the Malagasy region

- Antenna filiform, consisting of 13 segments.
- Scape not reaching posterior margin of head.
- Mesopleural oblique furrow usually vestigial, and when present, reaching pronotum far from pronotal posteroventral margin.
- Abdominal segment II broadly and dorsally attached to abdominal segment III.
- Abdominal segment II much smaller than segment III in lateral view.
- Protibia with one spur.
- Metatibia with one or two spurs.

Remarks. Our key includes five Amblyoponinae genera recorded from the Malagasy region. Key modified from Yoshimura and Fisher (2012).

### ﻿Male-based key to genera of the subfamily Amblyoponinae

**Table:** 

1	A single tibial spur present on metatibia (Fig. 10A). Mandible with apical and pre-apical teeth. Pterostigma reduced in size	***Prionopelta***
–	Two tibial spurs present on metatibia (Fig. 10B). Mandible with a single apical tooth. Pterostigma well developed	**2**
2	Constriction between abdominal segment II and abdominal segment III indistinct in dorsal view. Cinctus between abdominal segment III and abdominal segment IV indistinct. On forewing, radial sector vein fails to reach costal margin and is disconnected from radius vein (Fig. 11A)	***Adetomyrma***
–	Constriction between abdominal segment II and abdominal segment III distinct in dorsal view. Cinctus between abdominal segment III and abdominal segment IV distinct and deep. On forewing, radial sector vein reaches costal margin and is connected with radius vein (Fig. 11B)	**3**
3	Pygostyles present (Fig. 12A)	***Stigmatomma***
–	Pygostyles absent (Fig. 12B)	**4**
4	Anterior margin of clypeus with tooth-like projections. Radial sector vein on forewing fully complete (Fig. 13A). Radius vein on hindwing present	***Mystrium***
–	Anterior margin of clypeus without tooth-like projections. Radial sector vein on forewing wholly or partially absent between M+Rs and 2r-rs (Fig. 13B). Radius vein on hindwing absent	***Xymmer***

**Figure 10.**
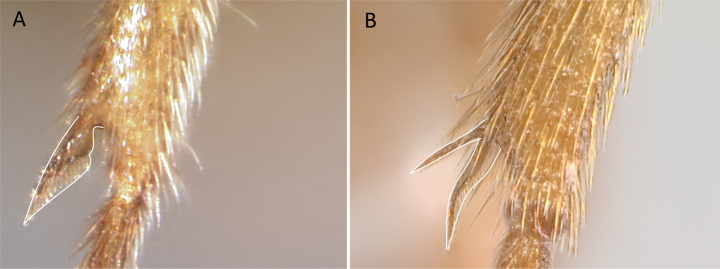
Tibial spur on metatibia **A***Prionopeltasubtilis* (CASENT0049809) **B***Mystriummirror* (CASENT0492154). Photographer Masashi Yoshimura.

**Figure 11.**
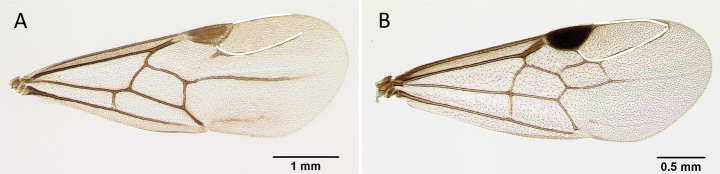
Venation of forewing **A***Adetomyrmacaputleae* (CASENT0218013) **B***Stigmatomma* mg01 (CASENT0083104). Photographer Masashi Yoshimura.

**Figure 12.**
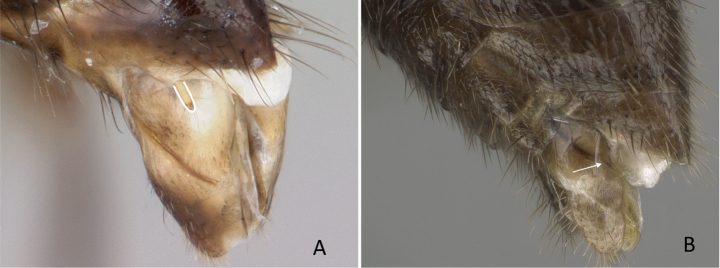
Posterior portion of abdomen in posterolateral view **A***Stigmatomma* mgm01 (CASENT0007139) **B***Xymmer* drm01 (CASENT0135825). Photographers April Nobile (**A**), Dimby Raharinjanahary (**B**).

**Figure 13.**
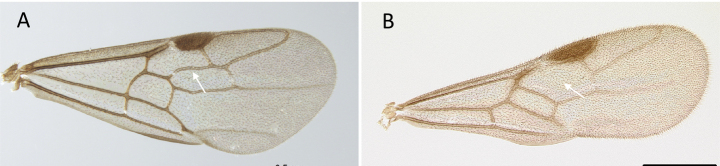
Venation of forewing **A***Mystriumbarrybressleri* (CASENT0078803) **B***Xymmer* mgm04 (CASENT0113147). Photographer Masashi Yoshimura.

#### *Adetomyrma* Ward, 1994

Antenna with 13 segments. Frontal carinae absent. Anterior margin of clypeus with tooth-like projections. Mandible falcate with single apical tooth. Palpal formula 3,3/2,3/2,2. Notauli absent for some species or distinct in *Adetomyrmagoblin*. Mesepimeron with or without epimeral lobe. Protibia with one spur. Mesotibia with two spurs. Metatibia with two spurs. In dorsal view, cinctus between abdominal segment III and abdominal segment IV indistinct. Pygostyles present. On forewing, pterostigma well developed. Costal vein (C) present. Cross-vein 1m-cu present. Radial sector vein (Rs) between M+Rs and 2r-rs wholly or partially absent and fails to reach costal margin. Cross-vein 2r-rs connected with radial sector vein posterior to pterostigma. Cross-vein 2rs-m absent. The cross-vein cu-a proximal to junction between media and cubitus vein. Media (M) fused with radial sector vein to form Rs+M. On hindwing, radius vein (R) absent. Radial sector vein (Rs) present. 1rs-m absent. The median vein, proximally fused with cubital vein (M+Cu), following separation continuing as a free abscissa (M). M+Cu present. 1rs-m+M absent. Free section of cubitus present. Cross-vein cu-a present.

#### *Mystrium* Roger, 1862

Antenna with 13 segments. Frontal carinae present. Anterior margin of clypeus with tooth-like projections. Mandible falcate with single apical tooth. Palpal formula 4,3. Notauli absent for some but distinct in *Mystriumrogeri*, *M.oberthueri*, *M.mysticum*, and *M.mirror*. Mesepimeron with epimeral lobe. Protibial with one spur. Mesotibia with single or two spurs. Metatibia with two spurs. In dorsal view, cinctus between abdominal segment III and abdominal segment IV distinct and deep. Pygostyles absent. On forewing; pterostigma well developed; costal vein (C) present, cross-vein 1m-cu present. Radial sector vein (Rs) fully present. Radial sector vein (Rs) reaches costal margin. Cross-vein 2r-rs connected with radial sector vein posterior to pterostigma. Cross-vein 2rs-m present. Cross-vein cu-a position variable located in line or proximal to junction between media and cubitus. Media (M) between Rs+M and 2rs-m completely present and after 2rs-m completely present. On hindwing, radius vein (R) present. Radial sector vein (Rs) present. 1rs-m present. The median vein (M), proximally fused with cubital vein (M+Cu), following separation continuing as a free abscissa (M) and joint apical to 1rs-m. M+Cu present. 1rs-m+M present. Free section of cubitus present. Cross-vein cu-a present.

#### *Prionopelta* Mayr, 1866

Antenna with 13 segments. Frontal carinae present. Anterior margin of clypeus with tooth-like projections. Mandible falcate with two sharp apical teeth. Palpal formula 2,2. Notauli present. Mesepimeron without epimeral lobe. Pro-, meso-, and metatibia with one spur. In dorsal view, cinctus between abdominal segment III and abdominal segment IV distinct and deep. Pygostyles present. On forewing, pterostigma reduced in size. Costal vein (C) present. Cross-vein 1m-cu present. Radial sector vein (Rs) absent between M+Rs and 2r-rs. Radial sector vein (Rs) reaches costal margin. Cross-vein 2r-rs connected with radial sector vein distal to pterostigma. Cross-vein 2rs-m present. Cross-vein cu-a proximal to junction between media and cubitus. Media (M) between Rs+M and 2rs-m completely present and after 2rs-m at least partially present. On hindwing, radius vein (R) present but absent in one species. Radial sector vein (Rs) present. 1rs-m present The median vein (M), proximally fused with cubital vein (M+Cu), following separation continuing as a free abscissa (M) and joint apical to 1rs-m. M+Cu present. 1rs-m+M present. Free section of cubitus absent. Cross-vein cu-a present.

#### *Stigmatomma* Roger, 1859

Antenna with 13 segments. Frontal carinae absent. Anterior margin of clypeus with tooth-like projections. Mandible falcate with single apical tooth. Palpal formula 4,3/4,2/3,2. Notauli present. Mesepimeron with epimeral lobe. Protibia with one spur. Mesotibia with one or two spurs. Metatibia with two spurs. In dorsal view, cinctus between abdominal segment III and abdominal segment IV distinct and deep. Pygostyles present. On forewing, pterostigma well developed. Costal vein (C) present. Cross-vein 1m-cu present. Radial sector vein (Rs) fully present. Radial sector vein (Rs) reaches costal margin. Cross-vein 2r-rs connected with radial sector vein posterior to pterostigma. Cross-vein 2rs-m present. Cross-vein cu-a located in line or proximal to junction between media and cubitus. Media (M) between Rs+M and 2rs-m completely present and after 2rs-m at least partially present. On hindwing, radius vein (R) present or absent. Radial sector vein (Rs) present. 1rs-m present. The median vein (M), proximally fused with cubital vein (M+Cu), following separation continuing as a free abscissa (M) and joint apical to 1rs-m. M+Cu present. 1rs-m+M present. Free section of cubitus present. Cross-vein cu-a present.

#### *Xymmer* Santschi, 1914

Antenna with 13 segments. Frontal carinae absent. Anterior margin of clypeus straight, without tooth-like projections. Mandible falcate with single apical tooth. Palpal formula 3,3 /3,2/4,3. Notauli present. Mesepimeron with epimeral lobe. Protibia with one spur. Mesotibia with one or without spur. Metatibia with two spurs. In dorsal view, cinctus between abdominal segment III and abdominal segment IV distinct and deep. Pygostyles absent. On forewing, pterostigma well developed. Costal vein (C) present. Cross-vein 1m-cu present. Radial sector vein (Rs) absent between M+Rs and 2r-rs. Radial sector vein (Rs) reaches costal margin. Cross-vein 2r-rs connected with radial sector vein posterior to pterostigma. Cross-vein 2rs-m present. Cross-vein cu-a proximal to junction between media and cubitus. Media (M) between Rs+M and 2rs-m completely present and after 2rs-m at least partially present. On hindwing, radius vein (R) absent. Radial sector vein (Rs) present. 1rs-m absent. The median vein (M), proximally fused with cubital vein (M+Cu). Media (M) absent and not fused apical to 1rs-m. M+Cu present. 1rs-m+M absent. Free section of cubitus absent. Cross-vein cu-a present.

##### ﻿DOLICHODERINAE Forel, 1878

Diagnosis of male ants of the subfamily Dolichoderinae in the Malagasy region

- Antenna filiform, consisting of 12 to 13 segments.
- Scape short, not reaching the posterior margin of head.
- Mesopleural oblique furrow reaching pronotum far away from pronotal posteroventral margin.
- Notauli absent.
- Scuto-scutellar suture simple.
- Single, well-developed spur presents on pro-, meso-, and metatibia.
- Abdominal segment II much smaller than segment III in lateral view.
- Abdominal segment II narrowly or broadly attached to abdominal segment III.
- No constriction present between abdominal segments III and IV.
- Jugal lobe absent.
- Pygostyles present.
- Wing venation: Venation on forewing varies. Radius vein (R), Sc+R+Rs, Radial sector vein (Rs), cubitus (Cu), anal (A), 2r-rs, and cu-a present in all genera. Media (M) present between Rs+M and 2rs-m. 2rs-m present or continuous with media. On hindwing, R+Rs and anal present. Radius vein and media apical to rs-m absent. M+Cu, cubitus, 1rs-m, and cu-a variable. Clavus moderate in size, and jugum absent.

Remarks. Our key includes six genera of Dolichoderinae recorded from the Malagasy region. Key modified from Yoshimura and Fisher (2011). It is crucial to acknowledge that although male specimens of *Ochetellusglaber* have yet to be collected in the Malagasy region, they have been incorporated into this key based on morphological traits observed in *O.glaber* collected from Japan. Notably, the genus *Linepithema*, with its species *L.humile*, has been recently reported in the Malagasy region (Reunion: Nève de Mévergnies et al. 2024), and has been incorporated into this key based on morphological traits observed in specimens collected in California, USA.

### ﻿Male-based key to genera of the subfamily Dolichoderinae

**Table:** 

1	Masticatory margin of mandible with many serrate denticles (Fig. 14A)	**2**
–	Masticatory margin of mandible with one to several large teeth (Fig. 14B)	**5**
2	On hindwing, M+Cu absent. In ventral view, telomere greatly expanded mesally, forming a distinct and more or less flat ventral face (Fig. 15A)	***Technomyrmex***
–	On hindwing, M+Cu present. In ventral view, telomere narrow, without a distinct ventral face (Fig. 15B)	**3**
3	With head in full-face view, scape long, reaching lower edge of lateral ocelli (Fig. 16A)	***Tapinoma***
–	With head in full-face view, scape short, not reaching lower edge of lateral ocelli (Fig. 16B)	**4**
4	With head in full-face view, second funicular segment shorter than scape and first funicular segment more cylindrical (Fig. 17A)	***Linepithema* (Reunion)**
–	With head in full-face view, second funicular segment longer than scape and first funicular segment conical (Fig. 17B)	***Aptinoma***
5	Mandible broadly spatulate, with a single, long, acute tooth on distal apex (Fig. 18A). Abdominal segment II narrowly attached to abdominal segment III	***Ravavy***
–	Mandible triangular, with several stout teeth on distal apex (Fig. 18B). Abdominal segment II broadly attached to abdominal segment III	***Ochetellus* (Mauritius, Reunion)**

**Figure 14.**
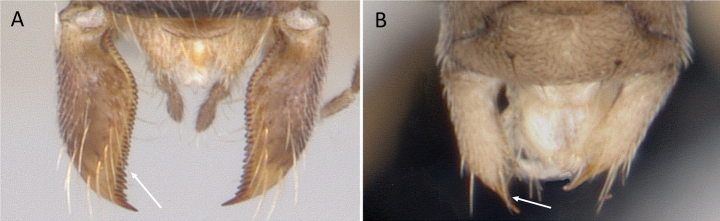
Mandible in full-face view **A***Technomyrmexdifficilis* (CASENT0049968) **B***Ravavymiafina* (CASENT0474633). Photographer April Nobile.

**Figure 15.**
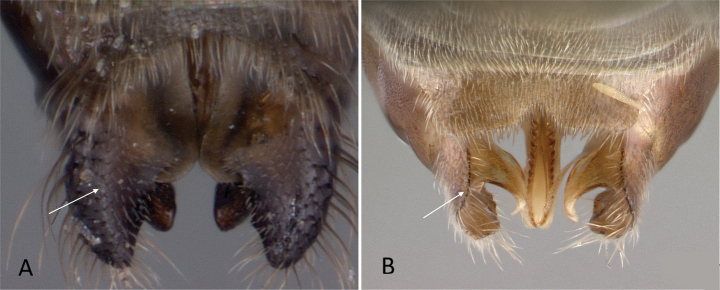
Telomere **A***Technomyrmex* mg08 (CASENT0049527) **B***Tapinoma* mg10 (CASENT0115650). Photographers Masashi Yoshimura (**A**), Erin Prado (**B**).

**Figure 16.**
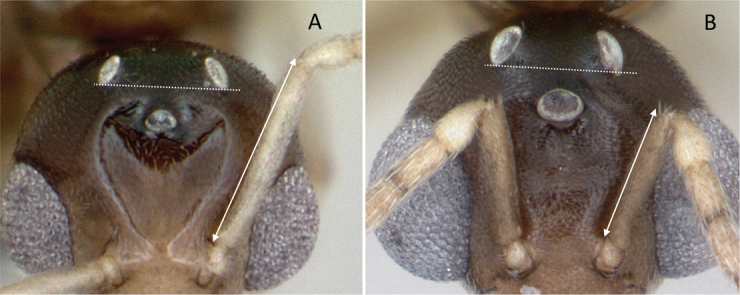
Head in full-face view showing the comparison of scape length **A***Tapinoma* mg12 (CASENT0115678) **B***Aptinomamangabe* (CASENT0173594). Photographer April Nobile.

**Figure 17.**
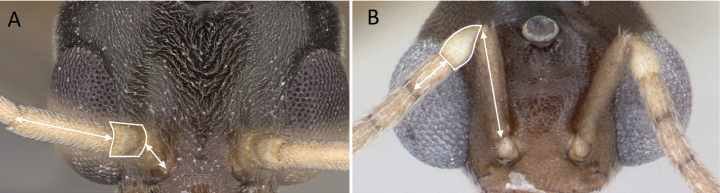
Head in full-face view, showing proportion of second funicular segment in relation to scape and form of first funicular segment **A***Linepithemahumile* (CASENT0724858) **B***Aptinomamangabe* (CASENT0173594) Photographers Wade Lee (**A**), April Nobile (**B**).

**Figure 18.**
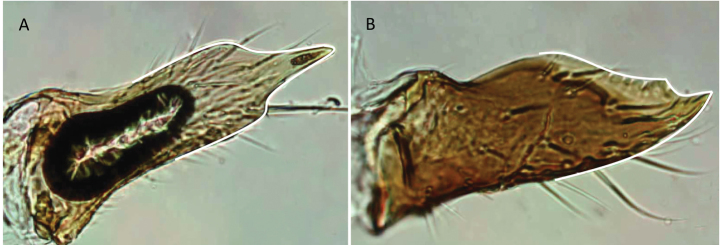
Mandible **A***Ravavymiafina* (CASENT0179530) **B***Ochetellusglaber* (CASENT0179489). Photographer Masashi Yoshimura.

#### *Aptinoma* Fisher, 2009

Antenna with 13 segments, scape shorter than 2+3 funicular segments, first funicular segment conical, second funicular segment straight. Medial hypostoma present. Mandible triangular, masticatory margin with serrate denticles. Palpal formula 6,3. Propodeal spiracle oval. Abdominal segment II not unusually expanded, narrowly attached to abdominal segment III. Abdominal segment III with a groove or indentation on anterior face. Pygostyles present. On forewing, pterostigma well developed; costal vein (C) and cross-vein 1m-cu present. Radial sector vein (Rs) partially absent between M+Rs and 2r-rs and reaches costal margin. Cross-vein 2r-rs connected to radial sector vein posterior to pterostigma. Cross-vein 2rs-m present. Cu-a proximal to junction between media and cubitus. Media between Rs+M and 2rs-m present. On hindwing, radius vein (R) present. Radial sector vein (Rs) present. Cross-vein 1rs-m absent. Media (M) absent. M+Cu present. 1rs-m+M absent. Free section of cubitus absent. Cross-vein cu-a present.

#### *Linepithema* Mayr, 1866

Antenna with 13 segments, scape shorter than second funicular segment, first funicular segment cylindrical, second funicular segment straight, last eight flagellar segments shorter. Mandible triangular, masticatory margin with serrate denticles. Basal margin of mandible smooth. Propodeal spiracle circular. Abdominal segment II squamiform and abdominal segment II narrowly attached to abdominal segment III. Pygostyles present. On forewing, pterostigma well developed; costal vein (C) and cross vein 1m-cu present. Radial sector vein (Rs) present between M+Rs and 2r-rs and reaches costal margin. Cross-vein 2r-rs connected with radial sector vein posterior to pterostigma. Media before junction of radial sector vein (Rs) present. Cu-a proximal to junction between media and cubitus. On hindwing, radial sector vein (Rs) present, 1rs-m+M present, M+Cu present, free section of cubitus Cu present.

#### *Ochetellus* Shattuck, 1992

Antenna with 12 segments. Scape shorter than 2+4 funicular segments. First funicular segment barrel-shaped. Second funicular segment straight. Medial hypostoma present. Mandible triangular, basal margin of mandible without denticles and smooth, and masticatory margin with several stout teeth and minute denticles (Yoshimura and Fisher 2011). Palpal formula 6,4. Propodeal spiracle circular. Abdominal segment II expanded laterally and widened dorsally, broadly attached to abdominal segment III. Abdominal segment III without a groove. Pygostyles present. On forewing, pterostigma well developed. Costal vein (C) and 1m-cu present. Radial sector vein (Rs) between M+Rs and 2r-rs complete and reaches costal margin. Cross-vein 2r-rs connected with radial sector vein posterior to pterostigma. Cross-vein 2rs-m present. Cu-a proximal to junction between media and cubitus. Media between Rs+M and 2rs-m completely absent. On hindwing, radius vein (R) present. Radial sector vein (Rs) present. Cross-vein 1rs-m absent. Media (M) absent. M+Cu usually present. 1rs-m+M present. Free section of cubitus present. Cross-vein cu-a present.

#### *Ravavy* Fisher, 2009

Antenna with 13 segments (Fisher 2009). Scape shorter than 2+5 funicular segments. First funicular segment conical. Second funicular segment bent laterally. Medial hypostoma absent. Mandible broadly spatulate, edentate. Palpal formula 6,3. Propodeal spiracle circular. Abdominal segment II not unusually expanded and narrowly attached to abdominal segment III. Abdominal segment III with a groove or indentation on anterior face. Pygostyles present. On forewing, pterostigma well developed. Costal vein (C) present. Cross-vein 1m-cu present. Radial sector vein (Rs) fused to M+Rs and reaches costal margin (Fisher 2009; Yoshimura and Fisher 2011). Cross-vein 2r-rs connected with radial sector vein posterior to pterostigma. Cross-vein 2rs-m absent. Cu-a proximal to junction between media and cubitus. Media before junction with Rs present. On hindwing, radius vein (R) present. Radial sector vein (Rs) present. Cross-vein 1rs-m absent. Media (M) absent. M+Cu present. 1rs-m+M present. Free section of cubitus absent. Cross-vein cu-a present.

#### *Tapinoma* Foerster, 1850

Antenna with 13 segments. Scape longer than 2+3 funicular segments but not exceeding posterior margin of head. First funicular segment conical. Second funicular segment straight. Medial hypostoma present. Mandible triangular, masticatory margin with or without serrate teeth. Palpal formula usually 6,4 but sometimes 6,3. Propodeal spiracle circular. Abdominal segment II not unusually expanded and narrowly attached to abdominal segment III. Abdominal segment III with a groove or indentation on anterior face. Pygostyles present. On forewing, pterostigma well developed. Costal vein (C) present. Cross-vein 1m-cu absent. Radial sector vein (Rs) fused to M+Rs. Radial sector vein (Rs) reaches costal margin. Cross-vein 2r-rs connected with radial sector vein posterior to pterostigma. Cross-vein 2rs-m absent. Cu-a proximal to junction between media and cubitus. Media between Rs+M and 2rs-m completely absent. On hindwing, radius vein (R) present. Radial sector vein (Rs) present. Cross-vein 1rs-m absent. Media (M) absent. M+Cu present. 1rs-m+M present. Free section of cubitus present. Cross-vein cu-a present.

#### *Technomyrmex* Mayr, 1872

Antenna with 13 segments. Scape shorter than 2+5 funicular segments. First funicular segment conical. Second funicular segment straight. Medial hypostoma present. Mandible triangular, masticatory margin of mandible wholly covered with serrate denticles. Palpal formula 6,4. Propodeal spiracle circular. Abdominal segment II not unusually expanded and narrowly attached to abdominal segment III. Abdominal segment III with a groove or indentation on anterior face. Pygostyles present. On forewing, pterostigma well developed. Costal vein (C) present. Cross-vein 1m-cu absent. Radial sector vein (Rs) fused to M+Rs. Radial sector vein (Rs) reaches costal margin. Cross-vein 2r-rs connected with radial sector vein posterior to pterostigma. Cross-vein 2rs-m absent. Cu-a proximal to junction between media and cubitus. Media between Rs+M and 2rs-m at least partially present. On hindwing, radius vein (R) absent. Radial sector vein (Rs) present. Cross-vein 1rs-m absent. Media (M) absent. M+Cu absent. 1rs-m+M absent. Free section of cubitus absent. Cross-vein cu-a absent.

##### ﻿DORYLINAE Leach, 1815

Diagnosis of male ants of the subfamily Dorylinae in the Malagasy region

- Antenna filiform, consisting of 11–13 segments.
- Scape not reaching posterior margin of head.
- Scuto-scutellar suture usually longitudinally sculptured.
- Abdominal segment II attached to abdominal segment III ventrally.
- Abdominal segment II much smaller than segment III in lateral view.
- Two distinct, long, narrow spines present on the posterior portion of abdominal sternum IX.
- Pygostyles absent.
- Protibia with one spur.
- Girdling constriction between pre- and post-sclerites of abdominal segments V and VI absent.

Remarks. Our key includes eight Dorylinae genera recorded from the Malagasy region. Key modified from Borowiec (2016). It is important to note that while the males of *Chrysapace* are currently unknown in the Malagasy region, they have been included in this key based on examination of SE Asian specimens.

### ﻿Male-based key to genera of the subfamily Dorylinae

**Table:** 

1	Antenna with 11 segments	***Ooceraea***
–	Antenna with 12 to13 segments	**2**
2	Maxillary palps very long and reaching occipital foramen, 6-segmented and visible in mounted specimens (Fig. 19A)	***Tanipone***
–	Maxillary palps short, never reaching occipital foramen, usually not visible without dissection and often with fewer than six segments (Fig. 19B)	**3**
3	Cross vein 2rs-m present complete in forewing (Fig. 20A). Mesotibiae with two tibial spurs	***Chrysapace***
–	Cross vein 2rs-m absent or at most stub-like in forewing (Fig. 20B). Mesotibiae with or without one tibial spur	**4**
4	Antenna with 12 segments. Mesotibiae without spurs (Fig. 21A)	***Simopone***
–	Antenna with 13 segments. Mesotibiae with a single spur, which may be simple and inconspicuous (Fig. 21B)	**5**
5	Costal vein (C) present in forewing (Fig. 22A)	**6**
–	Costal vein (C) absent in forewing (Fig. 22B)	**7**
6	Helcium circumference large and in profile dorsal surface of helcium arises from immediately below anterior dorsal angle of abdominal segment III (Fig. 23A). On forewing, radius vein (R) past pterostigma absent	***Lividopone***
–	Helcium circumference small and in profile dorsal surface of helcium arises some distance below anterodorsal angle of abdominal segment III (Fig. 23B). On forewing, radius vein (R) past pterostigma present	***Eburopone***
7	On forewing, radial sector vein partially absent between M+Rs and 2r-rs and not reaching costal margin; radius vein (R) absent on costal margin (Fig. 24A). Parafrontal ridges absent	***Lioponera***
–	On forewing, complete and not reaching costal margin; radius vein (R) absent on costal margin (Fig. 24B). Parafrontal ridges present	***Parasyscia***

**Figure 19.**
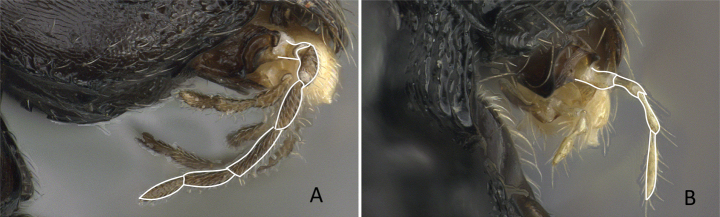
Maxillary palps **A***Taniponezona* (CASENT0168822) **B***Lividopone* mg10 (CASENT0027622). Photographer Michele Esposito.

**Figure 20.**
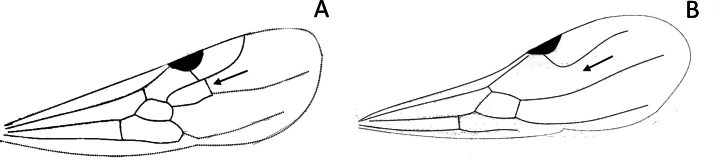
Forewing showing cross vein 2rs-m **A***Chrysapacesauteri* (CASENT0179567) **B***Eburopone* dr03 (CASENT0138666).

**Figure 21.**
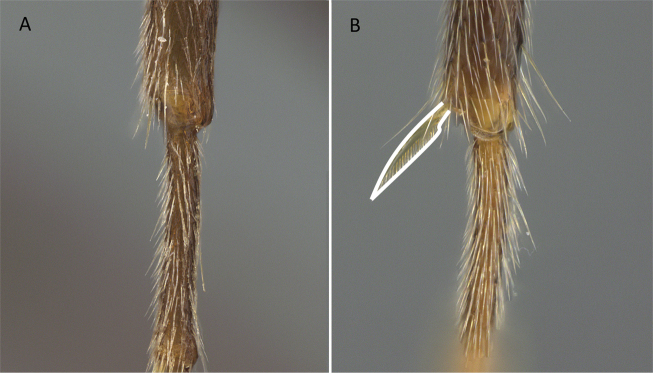
Tibial spurs on mesotibia **A***Simoponesilens* (CASENT0740895) **B***Lividopone* mg10 (CASENT0496142). Photographer Michele Esposito.

**Figure 22.**
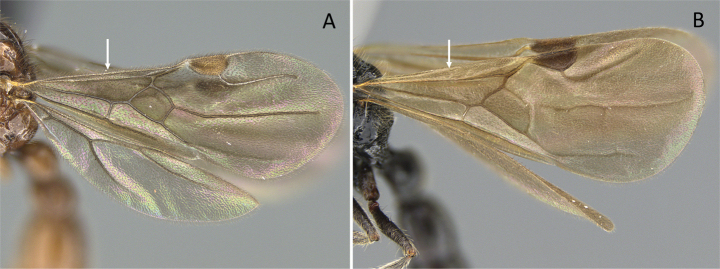
Forewing in lateral view showing costal vein (C) **A***Eburopone* dr03 (CASENT0138666) **B***Lioponera* mg06 (CASENT0138558). Photographer Michele Esposito.

**Figure 23.**
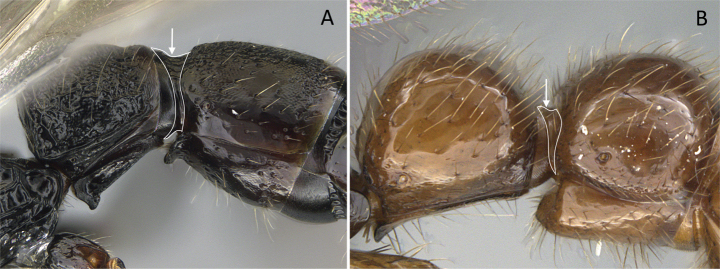
Abdominal segment II and III in lateral view showing helcium circumference **A***Lividopone* dr02 (CASENT0135633) **B***Eburopone* dr03 (CASENT0138666). Photographer Michele Esposito.

**Figure 24.**
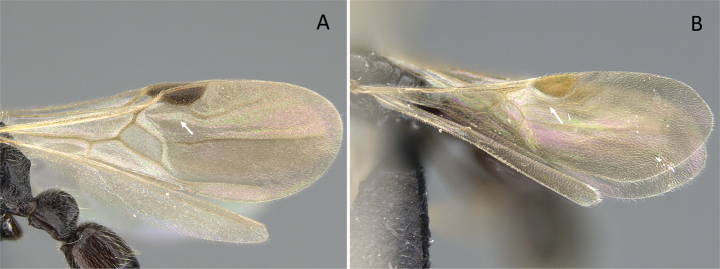
Forewing showing Rs vein **A***Lioponera* dr02 (CASENT0144823) **B***Parasysciaimerinensis* (CASENT0117837). Photographer Michele Esposito.

#### *Chrysapace* Crawley, 1924

Antenna with 13 segments. Clypeus without cuticular apron. Parafrontal ridges present. Torulo-posttorular complex vertical. Maxillary palps unknown. Labial palps unknown. Mandibles triangular, masticatory margin edentate. Ventrolateral margins of head without lamella or ridge extending towards mandibles and beyond carina surrounding occipital foramen. Carina surrounding occipital foramen unknown. Pronotal ﬂange separated from collar by distinct ridge. Notauli present. Transverse groove dividing mesopleuron present. Propodeal declivity with distinct dorsal edge or margin. Metapleural gland opening present. Propodeal spiracle present. Abdominal segment II anterodorsally marginate, dorsolaterally immarginate, and laterally above spiracle marginate. In profile dorsal surface of helcium arises some distance below anterodorsal angle of abdominal segment III. Prora forming a V-shaped protrusion. Spiracle openings of abdominal segments IV–VI circular. Mesotibia with two pectinate spurs. Metatibia with two pectinate spurs. Metatibial gland absent. Hind pretarsal claws with a tooth. On forewing, pterostigma broad. Costal vein (C) present. Radius vein (R) present. Radial sector vein (Rs) fully present between M+Rs and 2r-rs. Radial sector vein (Rs) fails to reach costal margin. Cross-vein 2r-rs present and connected with radial sector vein posterior to pterostigma. Cross-vein 2rs-m present (Borowiec 2016). Media (M) present, reaches wing margin. Cross-vein 1m-cu present. Cross-vein cu-a proximal to junction between media and cubitus. On hindwing, costal vein (C) absent. Radius vein (R) absent. Vein Sc+R present. Radial sector vein (Rs) present, not reaching wing margin. Cross-vein 1rs-m fused with M. Vein M+Cu present. Abscissa M present. Cross-vein cu-a present. Free section of cubitus present.

#### *Eburopone* Borowiec, 2016

Antenna with 13 segments. Clypeus with or without cuticular apron. Parafrontal ridges absent. Torulo-posttorular complex vertical. Maxillary palps 3- or 4-segmented. Labial palps 2- or 3-segmented. Mandibles triangular. Masticatory margin with teeth or falcate. Ventrolateral margins of head without lamella or ridge extending towards mandibles and beyond carina surrounding occipital foramen. Carina surrounding occipital foramen ventrally absent or present. Pronotal ﬂange not separated from collar by distinct ridge. Notauli present at least anteriorly, very rarely absent. Transverse groove dividing mesopleuron absent or present. Propodeal declivity reduced, without distinct dorsal edge or margin. Metapleural gland opening absent. Propodeal spiracle present. Abdominal segment II anterodorsally immarginate or marginate, dorsolaterally immarginate, and laterally above spiracle immarginate. In profile dorsal surface of helcium arises some distance below anterodorsal angle of abdominal segment III. Prora simple, not delimited by carina. Spiracle openings of abdominal segments IV–VI circular. Mesotibia with single pectinate spur. Metatibia with single pectinate spur. Metatibial gland present as oval patch of whitish cuticle. Hind pretarsal claws simple. On forewing, pterostigma broad. Costal vein (C) present. Radius vein (R) present. Radial sector vein (Rs) absent between M+Rs and 2r-rs. Radial sector vein (Rs) fails to reach costal margin. Cross-vein 2r-rs present, forming base of “free stigma vein.” Cross-vein 2rs-m absent. Media (M) reaches wing margin or not, rarely entirely absent. Cross-vein 1m-cu present or rarely absent. Cross-vein cu-a proximal to junction between media and cubitus. On hindwing, costal vein (C) absent. Radius vein (R) present, extending past Sc+R but not reaching distal wing margin. Vein Sc+R absent or present. Radial sector vein (Rs) absent or present, not reaching wing margin. Cross-vein 1rs-m fused with M or absent. Vein M+Cu absent or present. Abscissa M absent. Cross-vein cu-a absent or present. Free section of cubitus absent or present.

#### *Lioponera* Mayr, 1879

Antenna with 13 segments. Clypeus with cuticular apron. Parafrontal ridges absent. Torulo-posttorular complex vertical. Maxillary palps 3-segmented. Labial palps 2-segmented. Mandibles triangular. Masticatory margin edentate. Ventrolateral margins of head with or without cuticular ridge extending towards mandibles and beyond carina surrounding occipital foramen. Carina surrounding occipital foramen ventrally absent. Pronotal ﬂange not separated from collar by distinct ridge. Notauli absent or present. Transverse groove dividing mesopleuron present. Propodeal declivity with distinct dorsal edge or margin. Metapleural gland opening present. Propodeal spiracle present. Abdominal segment II anterodorsally immarginate or marginate, dorsolaterally marginate, and laterally above spiracle marginate. In profile dorsal surface of helcium arises some distance below anterodorsal angle of abdominal segment III. Prora forming a simple U-shaped margin or protrusion. Spiracle openings of abdominal segments IV–VI circular. Mesotibia with single pectinate spur. Metatibia with single pectinate spur. Metatibial gland absent. Hind pretarsal claws simple. On forewing, pterostigma broad. Costal vein (C) absent. Radius vein (R) absent. Radial sector vein (Rs) absent between M+Rs and 2r-rs. Radial sector vein (Rs) fails to reach costal margin. Cross-vein 2r-rs most often present and forming base of “free stigma vein.” Cross-vein 2rs-m absent. Media (M) fails to reach wing margin. Cross-vein 1m-cu present or more rarely absent. Cross-vein cu-a located close to junction between media and cubitus. On hindwing, costal vein (C) absent. Radius vein (R) absent. Vein Sc+R present. Radial sector vein (Rs) absent or present, not reaching wing margin. Cross-vein 1rs-m absent or present, approx. as long as M. Vein M+Cu absent or present. Abscissa M absent. Cross-vein cu-a absent or present. Free section of cubitus absent or present.

#### *Lividopone* Bolton & Fisher, 2016

Antenna with 13 segments. Clypeus with cuticular apron. Parafrontal ridges present. Torulo-posttorular complex vertical. Maxillary palps unknown. Labial palps unknown. Mandibles triangular. Masticatory margin edentate. Ventrolateral margins of head with cuticular ridge extending towards mandibles and beyond carina surrounding occipital foramen. Carina surrounding occipital foramen unknown. Pronotal ﬂange separated from collar by distinct ridge. Notauli present. Transverse groove dividing mesopleuron present. Propodeal declivity with distinct dorsal edge or margin. Metapleural gland opening absent. Propodeal spiracle present. Abdominal segment II anterodorsally marginate, dorsolaterally immarginate, and laterally above spiracle marginate. In profile dorsal surface of helcium arises from immediately below anterior dorsal angle of abdominal segment III prora forming a U-shaped protrusion. Spiracle openings of abdominal segments IV–VI circular. Mesotibia with single pectinate spur. Metatibia with single pectinate spur. Metatibial gland absent. Hind pretarsal claws simple. On forewing, pterostigma broad. Costal vein (C) absent. Radius vein (R) absent. Radial sector vein (Rs) fully present between M+Rs and 2r-rs. Radial sector vein (Rs) fails to reach costal margin. Cross-vein 2r-rs absent or present, forming base of “free stigma vein.” Cross-vein 2rs-m absent. Media (M) absent or a stub. Cross-vein 1m-cu absent or present. Cross-vein cu-a proximal to junction between media and cubitus. On hindwing, costal vein (C) absent. Radius vein (R) absent. Vein Sc+R absent. Radial sector vein (Rs) absent or stub present. Cross-vein 1rs-m absent or present, approx. as long as M. Vein M+Cu absent or present. Abscissa M absent or present. Cross-vein cu-a absent. Free section of cubitus absent or present.

#### *Ooceraea* Roger, 1862

Antenna with 11 segments. Clypeus with cuticular apron. Parafrontal ridges absent. Torulo-posttorular complex vertical. Maxillary palps 5-segmented. Labial palps 3-segmented. Mandibles triangular. Masticatory margin edentate. Ventrolateral margins of head without lamella or ridge extending towards mandibles and beyond carina surrounding occipital foramen. Carina surrounding occipital foramen ventrally absent. Pronotal ﬂange not separated from collar by distinct ridge, occasionally ridge marked on sides. Notauli present. Transverse groove dividing mesopleuron present. Propodeal declivity reduced, with or without distinct dorsal edge or margin. Metapleural gland opening absent. Propodeal spiracle present. Abdominal segment II anterodorsally immarginate, dorsolaterally immarginate, and laterally above spiracle marginate, inconspicuously in small species. In profile dorsal surface of helcium arises some distance below anterodorsal angle of abdominal segment III prora forming a simple U-shaped margin or a U-shaped margin with median ridge. Spiracle openings of abdominal segments IV–VI circular. Mesotibia with single pectinate spur. Metatibia with single pectinate spur. Metatibial gland present as oval patch of whitish cuticle. Hind pretarsal claws simple. On forewing, pterostigma broad. Costal vein (C) present or absent. Radius vein (R) absent. Radial sector vein (Rs) absent between M+Rs and 2r-rs. Radial sector vein (Rs) fails to reach costal margin. Cross-vein 2r-rs present, forming base of “free stigma vein.” Cross-vein 2rs-m absent. Media (M) fails to reach wing margin. Cross-vein 1m-cu absent or present. Cross-vein cu-a proximal to junction between media and cubitus. On hindwing, costal vein (C) absent. Radius vein (R) absent or present, extending past Sc+R but not reaching distal wing margin. Vein Sc+R absent, Vein Sc+R present. Radial sector vein (Rs) absent or present, not reaching wing margin. Cross-vein 1rs-m absent. Vein M+Cu absent or present. Abscissa M absent. Cross-vein cu-a absent or present. Free section of cubitus absent.

#### *Parasyscia* Emery, 1882

Antenna with 13 segments. Clypeus with cuticular apron. Parafrontal ridges present. Torulo- posttorular complex vertical. Maxillary palps 2-segmented. Labial palps 2-segmented. Mandibles triangular. Masticatory margin edentate. Ventrolateral margins of head without lamella or ridge extending towards mandibles and beyond carina surrounding occipital foramen. Carina surrounding occipital foramen ventrally absent. Pronotal ﬂange separated from collar by distinct ridge mostly on sides or not separated. Notauli absent or present. Transverse groove dividing mesopleuron present. Propodeal declivity reduced, with or without distinct dorsal edge or margin. Metapleural gland opening absent. Propodeal spiracle present. Abdominal segment II anterodorsally immarginate or marginate, dorsolaterally immarginate, and laterally above spiracle marginate. In profile dorsal surface of helcium arises some distance below anterodorsal angle of abdominal segment III. Prora forming a U-shaped margin with median ridge. Spiracle openings of abdominal segments IV–VI circular. Mesotibia with single pectinate spur. Metatibia with single pectinate spur. Metatibial gland absent. Hind pretarsal claws simple. On forewing, pterostigma broad. Costal vein (C) absent. Radius vein (R) absent. Radial sector vein (Rs) partially absent between M+Rs and 2r-rs. Radial sector vein (Rs) fails to reach costal margin. Cross-vein 2r-rs present and connected with radial sector vein posterior to pterostigma. Cross-vein 2rs-m absent. Media (M) fails to reach wing margin. Cross-vein 1m-cu absent or present. Cross-vein cu-a located close to junction between media and cubitus. On hindwing, costal vein (C) absent. Radius vein (R) absent. Vein Sc+R absent. Radial sector vein (Rs) present, not reaching wing margin. Cross-vein 1rs-m present, approx. as long as M. Vein M+Cu present. Abscissa M absent or present. Cross-vein cu-a present. Free section of cubitus present.

#### *Simopone* Forel, 1891

Antenna with 12 segments. Clypeus without cuticular apron. Parafrontal ridges present. Torulo- posttorular complex horizontal. Maxillary palps 5- or 6-segmented. Labial palps 3- or 4-segmented. Mandibles triangular. Masticatory margin edentate. Ventrolateral margins of head without lamella or ridge extending towards mandibles and beyond carina surrounding occipital foramen. Carina surrounding occipital foramen ventrally absent. Pronotal ﬂange separated from collar by distinct ridge. Notauli present. Transverse groove dividing mesopleuron absent. Propodeal declivity with distinct dorsal edge or margin. Metapleural gland opening absent. Propodeal spiracle present. Abdominal segment II anterodorsally marginate, dorsolaterally immarginate, and laterally above spiracle marginate. In profile dorsal surface of helcium arises some distance below anterodorsal angle of abdominal segment III. Prora forming a U-shaped protrusion. Spiracle openings of abdominal segments IV–VI circular. Mesotibia without spurs. Metatibia with single pectinate spur. Metatibial gland absent. Hind pretarsal claws with a tooth. On forewing, pterostigma broad. Costal vein (C) absent. Radius vein (R) absent. Radial sector vein (Rs) fully present between M+Rs and 2r-rs. Radial sector vein (Rs) fails to reach costal margin. Cross-vein 2r-rs present and connected with radial sector vein posterior to pterostigma. Cross-vein 2rs-m absent. Media (M) reaches to wing margin. Cross-vein 1m-cu present or absent. Cross-vein cu-a proximal to junction between media and cubitus. On hindwing, costal vein (C) absent. Radius vein (R) absent. Vein Sc+R present. Radial sector vein (Rs) absent. Cross-vein 1rs-m present, approx. as long as M, never tubular. Vein M+Cu present. Abscissa M present. Cross-vein cu-a present. Free section of cubitus present.

#### *Tanipone* Bolton & Fisher, 2012

Antenna with 13 segments. Clypeus without cuticular apron. Parafrontal ridges absent. Torulo- posttorular complex vertical. Maxillary palps 6-segmented. Labial palps 4-segmented. Mandibles triangular. Masticatory margin edentate. Ventrolateral margins of head without lamella or ridge extending towards mandibles and beyond carina surrounding occipital foramen. Carina surrounding occipital foramen ventrally present. Pronotal ﬂange separated from collar by distinct ridge or not. Notauli absent. Transverse groove dividing mesopleuron present. Propodeal declivity with distinct dorsal edge or margin. Metapleural gland opening absent. Propodeal spiracle present. Abdominal segment II anterodorsally immarginate, dorsolaterally immarginate, and laterally above spiracle marginate. In profile dorsal surface of helcium arises some distance below anterodorsal angle of abdominal segment III. Prora forming a simple U-shaped margin or U-shaped protrusion. Spiracle openings of abdominal segments IV–VI circular. Mesotibia without spurs. Metatibia with single pectinate spur. Metatibial gland absent. Hind pretarsal claws with a tooth. On forewing, pterostigma broad. Costal vein (C) absent. Radius vein (R) absent. Radial sector vein (Rs) absent between M+Rs and 2r-rs. Radial sector vein (Rs) fails to reach to costal margin. Cross-vein 2r-rs absent or present and forming base of “free” stigmal vein. Cross-vein 2rs-m absent. Media (M) absent or present, reaches to wing margin. Cross-vein 1m-cu absent or present. Cross-vein cu-a proximal to junction media. On hindwing, costal vein (C) absent. Radius vein (R) absent. Vein Sc+R present. Radial sector vein (Rs) absent or present, reaching wing margin. Cross-vein 1rs-m absent or present, approx. as long as M. Vein M+Cu present. Abscissa M absent. Crossvein cu-a absent or present. Free section of cubitus present.

##### ﻿FORMICINAE Latreille, 1809

Diagnosis of male ants of the subfamily Formicinae in the Malagasy region

- Antenna filiform, consisting of 10–13 segments.
- Scape reaching or exceeding posterior margin of head.
- Mesopleural oblique furrow reaching pronotum far from pronotal posteroventral margin.
- Scuto-scutellar suture simple.
- Abdominal segment II attached to abdominal segment III ventrally.
- Abdominal segment II much smaller than segment III in lateral view.
- Apical portion of abdominal sternum IX not bi-spinose.
- Pygostyles well developed.
- Metatibia with one spur.

Remarks. Our article provides a guide highlighting nine genera of male Formicinae ants found in the Malagasy region.

### ﻿Male-based key to genera of the subfamily Formicinae

**Table:** 

1	Antenna with 10 segments, maxillary palp formula always 5,3 (Fig. 25A)	***Brachymyrmex***
–	Antenna with 12 or 13 segments, maxillary palp formula 6,4 (Fig. 25B)	**2**
2	Antenna with 12 segments	**3**
–	Antenna with 13 segments	**6**
3	Masticatory margin of mandible with 8 or 9 denticles (Fig. 26A)	***Anoplolepis* (Seychelles)**
–	Masticatory margin of mandible with < 5 denticles (Fig. 26B)	**4**
4	Funiculus longer than mesosoma length (Fig. 27A)	***Tapinolepis***
–	Funiculus shorter than mesosoma length (Fig. 27B)	**5**
5	First funicular segment length only slightly greater than that of second funicular segment in medial view. Malar space well developed, approx. as wide as scape width (Fig. 28A). Maxillary palp longer than maximum eye length	***Lepisiota***
–	First funicular segment length ~ 3× that of second funicular segment in medial view. Malar space extremely reduced, much narrower than scape width (Fig. 28B). Maxillary palp shorter than maximum eye length	***Plagiolepis***
6	Paired coarse setae absent from frons (Fig. 29A). Aroliae hypertrophied, conspicuous. Funiculus shorter than mesosomal length	***Camponotus***
–	Paired coarse setae present on frons (Fig. 29B). Aroliae small, inconspicuous. Funiculus longer than mesosoma length	**7**
7	Scape with standing macrosetae (Fig. 30A)	**8**
–	Scape lacking standing macrosetae (Fig. 30B)	**9**
8	In lateral view, first funicular segment distinctly longer than second funicular segment (Fig. 31A)	***Nylanderia***
–	In lateral view, first funicular segment shorter than or equal to second funicular segment (Fig. 31B)	***Paratrechina***
9	Scape slightly shorter than head length (Fig. 32A). Maxillary palp longer than head length	***Paraparatrechina***
–	Scape much longer than head length (Fig. 32B). Maxillary palp approx. as long as head length	***Paratrechinalongicornis***

**Figure 25.**
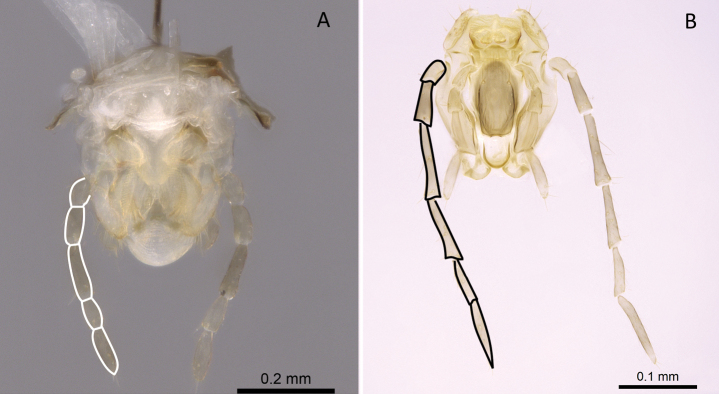
Maxillary palp **A***Brachymyrmexcordemoyi* (CASENT0740909) **B***Tapinolepis* mg01 (CASENT0763590). Photographer Veronica M. Sinotte.

**Figure 26.**
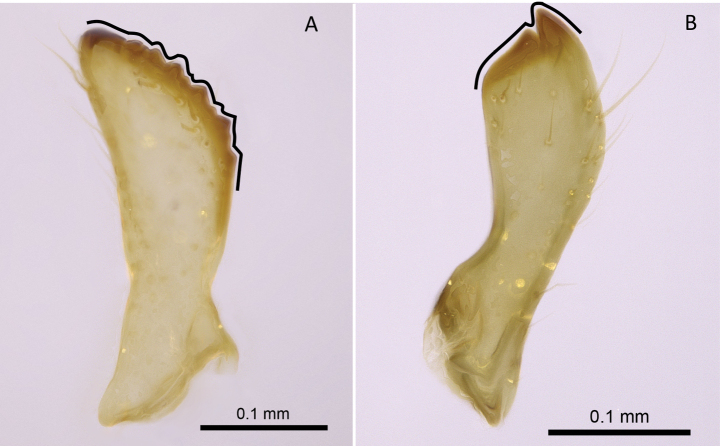
Mandible showing the number of teeth on the masticatory margin **A***Anoplolepisgracilipes* (CASENT0158950) **B***Nylanderiaamblyops* (CASENT0740913). Photographer Veronica M. Sinotte.

**Figure 27.**
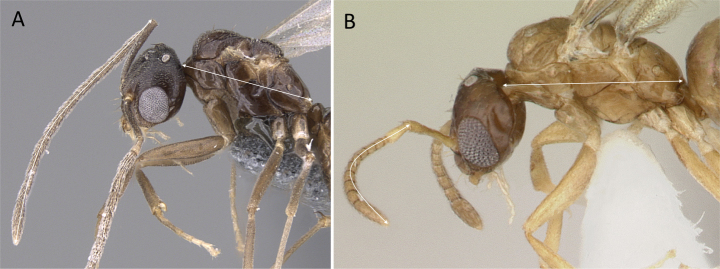
Body in lateral view comparing the length of the funiculus and mesosoma **A***Tapinolepis* mg01 (CASENT0763590) **B***Plagiolepis* mg02 (CASENT0179486). Photographers Veronica M. Sinotte (**A**), Erin Prado (**B**).

**Figure 28.**
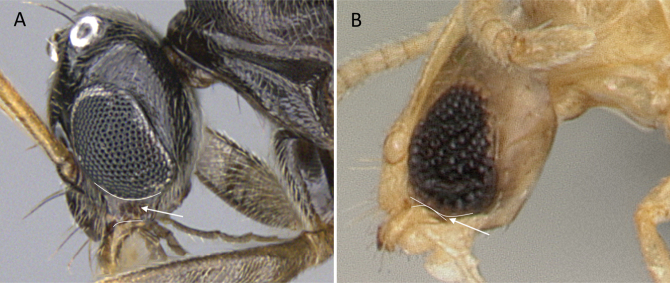
Head in lateral view showing the size of the malar space **A***Lepisiotacapensis* (CASENT0861517) **B***Plagiolepisalluaudi* (CASENT0495472). Photographers Michele Esposito (**A**), Erin Prado (**B**).

**Figure 29.**
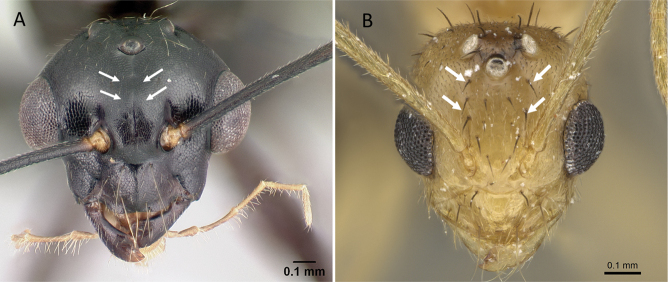
Head in full-face view showing setae disposition of frons **A***Camponotusalamaina* (CASENT0481800) **B***Nylanderiaamblyops* (CASENT0066704). Photographers Erin Prado (**A**), Michele Esposito (**B**).

**Figure 30.**
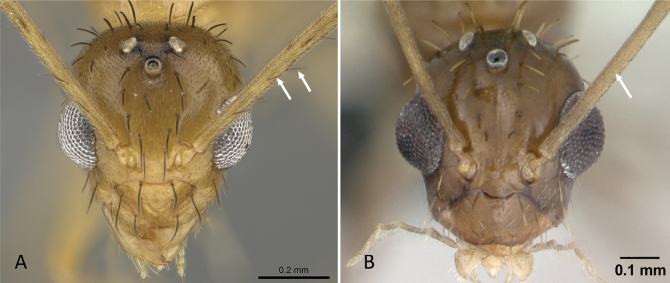
In full-face view, scape **A***Nylanderia* jsl-galo (CASENT0370667) **B***Paratrechinalongicornis* (CASENT0137341). Photographers Michele Esposito (**A**), Erin Prado (**B**).

**Figure 31.**
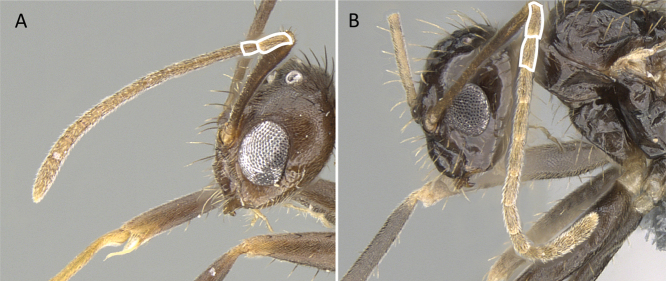
Antennae in lateral view comparing length of first funicular segment and second funicular segment of funiculus **A***Nylanderiabourbonica* (CASENT0160276) **B***Paratrechinaankarana* (CASENT0701215). Photographer Michele Esposito.

**Figure 32.**
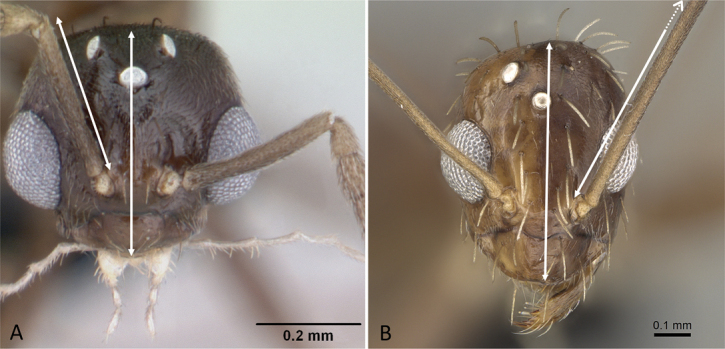
Head in full-face view comparing length of scape and head **A***Paraparatrechinaglabra* (CASENT0497708) **B***Paratrechinalongicornis* (CASENT0244951). Photographers April Nobile (**A**), Michele Esposito (**B**).

#### *Anoplolepis* Santschi, 1914

Antenna with 12 segments. Scape distinctly longer than head. Scape lacking standing setae. First funicular segment slightly shorter than second funicular segment in medial view. Funiculus subequal in length to mesosoma. Mandibles well developed, masticatory margin of mandible with eight or nine denticles. Palpal formula 6,4; maxillary palp exceeding hypostomal margin, but not reaching occipital foramen. Frons lacking paired coarse setae. Malar space well developed, broader than maximum scape width. Propodeal spiracle slit-shaped. Abdominal segment II lacking peduncle, node well developed. On forewing, pterostigma reduced in size. Costal vein (C) present. Cross-vein 1m-cu absent. Radial sector vein (Rs) fused to M+Rs. Radial sector vein (Rs) reaches costal margin. Cross-vein 2r-rs connected with radial sector vein posterior to pterostigma. Cross-vein 2rs-m absent. Cross-vein cu-a proximal to junction between media and cubitus. Media (M) fails to reach wing margin. On hindwing, radius vein (R) present. Radial sector vein (Rs) present. 1rs-m absent. Media (M) present. M+Cu present. 1rs-m+M present. Free section of cubitus absent. Cross-vein cu-a absent. Aroliae small, inconspicuous.

#### *Brachymyrmex* Mayr, 1868

Antenna with 10 segments. Aroliae small, inconspicuous. Mandibles reduced, spatulate to spiniform. Masticatory margin of mandible uni- to bidentate. Palpal formula 5,3. Maxillary palp approx. as long as maximum eye diameter. Frons lacking paired coarse setae. Scape shorter than head length. Scape lacking standing macrosetae. First funicular segment slightly longer than second funicular segment in medial view. Funiculus shorter than mesosoma length. Malar space well developed, approx. as long as scape is wide. Propodeal spiracle circular. Abdominal segment II lacking peduncle and node, very short anteroposteriorly. On forewing, pterostigma well developed. Costal vein (C) absent. Cross-vein 1m-cu absent. Radial sector vein (Rs) fused to M+Rs. Radial sector vein (Rs) fails to reach costal margin. Cross-vein 2r-rs connected with radial sector vein posterior to pterostigma. Cross-vein 2rs-m absent. Cross-vein cu-a proximal to junction between media and cubitus. Media (M) fails to reach wing margin. On hindwing, veins present, 1rs-m incomplete.

#### *Camponotus* Mayr, 1861

Antenna with 13 segments. Aroliae hypertrophied, conspicuous. Mandibles well developed, lobate. Masticatory margin of mandible without or with one denticle. Palpal formula 6,4. Maxillary palp exceeding hypostomal margin, exceeding or occipital foramen or not. Frons lacking paired coarse setae. Scape subequal to longer than head length. Scape with or without standing setae. First funicular segment longer or shorter than second funicular segment in medial view. Funiculus shorter than mesosomal length. Malar space well developed, much broader than maximum scape width. Propodeal spiracle slit-shaped. Abdominal segment II lacking long peduncle, node well developed. On forewing, pterostigma well developed. Costal vein (C) present. Cross-vein 1m-cu absent. Radial sector vein (Rs) fused to M+Rs. Radial sector vein (Rs) reaches to costal margin. Cross-vein 2r-rs connected with radial sector vein posterior to pterostigma. Cross-vein 2rs-m absent. Cu-a proximal to junction between media and cubitus. Media (M) fails to reach wing margin. On hindwing, radius vein (R) absent. Radial sector vein (Rs) present. 1rs-m absent. Media (M) present. M+Cu present. 1rs-m+M present. Free section of cubitus present. Cross-vein cu-a present.

#### *Lepisiota* Santschi, 1926

Male description based on male of *Lepisiotacapensis* Mayr, 1862. *Lepisiotabipartita* Smith, 1861is known from Réunion but males have not yet been collected.

Antenna with 12 segments. Aroliae small, inconspicuous. Ocelli placed close to occipital margin in front view. Anteromedian margin of clypeus straight. Mandibles well developed. Masticatory margin of mandible with four denticles. Palpal formula 6,4. Maxillary palp approx. as long as head length. Frons lacking paired coarse setae. Scape slightly longer than head length. Scape lacking standing macrosetae. First funicular segment subequal to or longer than second funicular segment in medial view. Funiculus shorter than mesosoma length. Malar space well developed, approx. as long as scape width. Propodeal spiracle oval. Abdominal segment II lacking peduncle and node, anteroposteriorly short. On forewing, pterostigma well developed. Costal vein (C) present. Cross-vein 1m-cu absent. Radial sector vein (Rs) fused to M+Rs. Radial sector vein (Rs) reaches costal margin. Cross-vein 2r-rs connected with radial sector vein posterior to pterostigma. Cross-vein 2rs-m absent. Cu-a proximal to junction between media and cubitus. Media (M) reaches wing margin. On hindwing, radius vein (R) absent. Radial sector vein (Rs) present. 1rs-m absent. Media (M) absent. M+Cu present. 1rs-m+M present. Free section of cubitus absent. Cross-vein cu-a present.

#### *Nylanderia* Emery, 1906

Antenna with 13 segments. Aroliae small, inconspicuous. Mandibles well developed. Masticatory margin of mandible with two denticles. Palpal formula 6,4. Maxillary palp longer than compound eye diameter and shorter than head length. Frons with paired coarse setae. Scape longer than head length but much shorter than mesosoma length. Scape usually with standing macrosetae. First funicular segment distinctly longer than second funicular segment in medial view. Funiculus longer than mesosoma length. Malar space very broad, approx. as long as first funicular segment. Propodeal spiracle circular. Abdominal segment II squamiform, posteriorly pedunculate. On forewing, pterostigma reduced in size. Costal vein (C) absent. Cross-vein 1m-cu absent. Radial sector vein (Rs) fused to M+Rs. Radial sector vein (Rs) reaches to costal margin. Cross-vein 2r-rs connected with radial sector vein posterior to pterostigma. Cross-vein 2rs-m absent. Cu-a proximal to junction between media and cubitus. Media (M) fails to reach wing margin. On hindwing, radius vein (R) absent. Radial sector vein (Rs) present. 1rs-m absent. Media (M) present. M+Cu present. 1rs-m+M present. Free section of cubitus present. Cross-vein cu-a present.

#### *Paraparatrechina* Donithorpe, 1947

Antenna with 13 segments. Aroliae small, inconspicuous. Mandibles well developed, spatulate. Masticatory margin of mandible with single apical tooth. Palpal formula 6,4. Maxillary palp longer than head length. Frons with paired coarse setae. Scape slightly shorter than head length. Scape lacking standing macrosetae. First funicular segment shorter than second funicular segment in medial view from. Funiculus longer than mesosoma length. Malar space broader than scape width. Propodeal spiracle circular. Abdominal segment II squamiform, posteriorly pedunculate. On forewing, pterostigma reduced in size. Costal vein (C) absent. Cross-vein 1m-cu absent. Radial sector vein (Rs) fused to M+Rs. Radial sector vein (Rs) reaches costal margin. Cross-vein 2r-rs connected with radial sector vein posterior to pterostigma. Cross-vein 2rs-m absent. Cross-vein cu-a proximal to junction between media and cubitus. Media (M) present and fails to reach wing margin. On hindwing, radius vein (R) absent. Radial sector vein (Rs) present. Cross-vein 1rs-m absent. Media (M) absent. M+Cu absent. 1rs-m+M present. Free section of cubitus absent. Cross-vein cu-a present.

#### *Paratrechina* Motschoulsky, 1863

*Paratrechinalongicornis* Latreille, 1802

Antenna with 13 segments. Aroliae small, inconspicuous. Mandibles well developed, spatulate. Masticatory margin of mandible with single apical tooth. Palpal formula 6,4. Maxillary palp approx. as long as head. Frons with paired coarse setae. Scape very long, longer than mesosoma. Scape lacking standing macrosetae. First funicular segment slightly shorter than second funicular segment in medial view. Funiculus longer than mesosoma length. Malar space very broad, approx. as long as first funicular segment. Propodeal spiracle circular. Abdominal segment II squamiform, posteriorly pedunculate. On forewing, pterostigma reduced in size. Costal vein (C) absent. Cross-vein 1m-cu absent. Radial sector vein (Rs) fused to M+Rs. Radial sector vein (Rs) reaches costal margin. Cross-vein 2r-rs connected with radial sector vein posterior to pterostigma. Cross-vein 2rs-m absent. Cu-a proximal to junction between media and cubitus. Media (M) present and fails to reach wing margin. On hindwing, radius vein (R) absent. Radial sector vein (Rs) present. Cross-vein 1rs-m absent. Media (M) absent. M+Cu absent. 1rs-m+M present. Free section of cubitus absent. Cross-vein cu-a present.

#### *Paratrechinaankarana* LaPolla & Fisher, 2014

Antenna with 13 segments. Aroliae small, inconspicuous. Mandibles well developed, spatulate. Masticatory margin of mandible with single apical tooth. Palpal formula 6,4. Maxillary palp approx. as long as head. Frons with paired coarse setae. Scape very long, longer than mesosoma. Scape usually with standing macrosetae. First funicular segment slightly shorter than second funicular segment in medial view. Funiculus longer than mesosoma length. Malar space very broad, approx. as long as first funicular segment. Propodeal spiracle circular. Abdominal segment II squamiform, posteriorly pedunculate. On forewing, pterostigma reduced in size. Costal vein (C) absent. Cross-vein 1m-cu absent. Radial sector vein (Rs) fused to M+Rs. Radial sector vein (Rs) reaches costal margin. Cross-vein 2r-rs connected with radial sector vein posterior to pterostigma. Cross-vein 2rs-m absent. Cross-vein cu-a proximal to junction between media and cubitus. Media (M) fails to reach wing margin. On hindwing, radius vein (R) absent. Radial sector vein (Rs) present. Cross-vein 1rs-m absent. Media (M) absent. M+Cu absent. 1rs-m+M present. Free section of cubitus absent. Cross-vein cu-a present.

*Paratrechinaantsingy* LaPolla & Fisher, 2014 the male is not known

#### *Plagiolepis* Mayr, 1861

Antenna with 12 segments. Aroliae small, inconspicuous. Mandibles well developed. Masticatory margin of mandible with two or three teeth. Palpal formula 6,4. Maxillary palp slightly longer than compound eye. Frons lacking paired coarse setae. Scape slightly longer than head. Scape lacking standing macrosetae. First funicular segment ~ 2× length of second funicular segment in medial view. Funiculus shorter than mesosoma length. Malar space reduced, shorter than scape width. Propodeal spiracle circular. Abdominal segment II anteroposteriorly short, posteriorly pedunculate. On forewing, pterostigma reduced in size. Costal vein (C) absent. Cross-vein 1m-cu absent. Radial sector vein (Rs) fused to M+Rs. Radial sector vein (Rs) reaches to costal margin. Cross-vein 2r-rs connected with radial sector vein posterior to pterostigma. Cross-vein 2rs-m absent. Cross-vein cu-a proximal to junction between media and cubitus. Media (M) fails to reach wing margin. On hindwing, radius vein (R) absent. Radial sector vein (Rs) present. Cross-vein 1rs-m absent. Media (M) absent. M+Cu absent. 1rs-m+M present. Free section of cubitus absent. Cross-vein cu-a present.

#### *Tapinolepis* Emery, 1925

Antenna with 12 segments. Aroliae small, inconspicuous. Mandibles well developed. Masticatory margin of mandible with four denticles. Palpal formula 6,4. Maxillary palp slightly shorter than head length. Frons lacking paired coarse setae. Scape slightly shorter than head length. Scape lacking standing macrosetae. First funicular segment shorter than second funicular segment in medial view. Funiculus longer than mesosoma. Malar space well developed, approx. as long as scape width. Propodeal spiracle circular. Abdominal segment II squamiform, lacking peduncle and with short node. On forewing, pterostigma well developed. Costal vein (C) present. Cross-vein 1m-cu absent. Radial sector vein (Rs) fused to M+Rs. Radial sector vein (Rs) reaches to costal margin. Cross-vein 2r-rs connected with radial sector vein posterior to pterostigma. Cross-vein 2rs-m absent. Cross-vein cu-a proximal to junction between media and cubitus. Media (M) fails to reach wing margin. On hindwing, radius vein (R) absent. Radial sector vein (Rs) present. 1rs-m absent. Media (M) absent. M+Cu present. 1rs-m+M present. Free section of cubitus absent. Cross-vein cu-a present.

##### ﻿MYRMICINAE Lepeletier de Saint-Fargeau, 1835

Diagnosis of male ants of the subfamily Myrmicinae in the Malagasy region

- Antenna filiform, consisting of 10 to 13 segments.
- Abdominal segment II attached to abdominal segment III ventrally.
- Abdominal segment II nearly as large or longer than III in lateral view
- Apical portion of abdominal sternum IX not bi-spinose.
- Pygostyles well developed.
- Front tibial with or without spur.
- Metatibia with one or spur absent.

Remarks. Our key includes thirty genera of male myrmicinae recorded from the Malagasy region. Males for *Dicroaspis* are not yet known from the Malagasy region and the diagnosis is based on males from the Afrotropics.

### ﻿Male-based key to genera of the subfamily Myrmicinae

**Table:** 

1	In profile, occipital carina strongly developed (Fig. 33A); mesoscutellum strongly elevated above metanotum; in dorsal view, scutellum smooth and convex (Fig. 33C). With head in full-face view, mandible always triangular	***Aphaenogaster* (Tribe Stenammini)**
–	In profile, occipital carina weakly developed (Fig. 33B); mesoscutellum slightly convex to flat; in dorsal view, scutellum with or without sculpture (Fig. 33D). With head in full-face view, mandible broadly triangular to reduced (spatulate or linear)	**2**
2	In profile, posterodorsal margin of head almost straight from base of lateral ocelli to midpoint of occipital carina (Fig. 34A)	**3 (Tribe Attini, part 1)**
–	In profile, posterodorsal margin of head gradually rounded from base of lateral ocelli to midpoint of occipital margin (Fig. 34B)	**5 (Tribe Attini, part2)**
3	Mandible with 3 teeth. Scape long, distinctly exceeding posterior margin of head in full-face view (Fig. 35A)	***Cyphomyrmex* (Reunion)**
–	Mandible edentate. Scape not reaching posterior margin of head in full-face view (Fig. 35B)	**4**
4	Radial sector vein on forewing is curved toward costal margin and reaches costal margin (Fig. 36A)	***Eurhopalothrix* (Comoros)**
–	Radial sector vein on forewing is downcurved and never reaches costal margin (Fig. 36A)	***Strumigenys***
5	Cross vein 2rs-m present on forewing (Fig. 37A)	***Pheidole***
–	Cross vein 2rs-m absent on forewing (Fig. 37B)	**6**
6	Mandible strongly developed; masticatory margin with 7 large teeth which increase in size from apex to base; between each tooth is a minute denticle (Fig. 38A)	***Pilotrochus***
–	Mandible normal to reduced; masticatory margin edentate to multidentate with many acute teeth which decrease in size from apex to base; without denticle between teeth (Fig. 38B)	**7**
7	In lateral view, anterior margin of promesonotum forms a continuous outline, pronotal furrow not breaking outline (Fig. 39A)	**8 (Tribe Solenopsidini)**
–	In lateral view, anterior margin of promesonotum interrupted by an impressed pronotal furrow that breaks outline (Fig. 39B) or mesonotum strongly produced anterodorsally (Fig. 39C)	**12 (Tribe Crematogastrinii)**
8	Antenna with 12 segments	***Solenopsis***
–	Antenna with 13 segments	**9**
9	In full-face view, first funicular segment subglobular; posteromedian margin of clypeus effaced so that clypeus and frons form a continuous surface (Fig. 40A); mandible triangular with distinct basal angle, masticatory margin with exactly 4 teeth	***Erromyrma***
–	In full-face view, first funicular segment not globular; posteromedian margin of clypeus visible (Fig. 40B); mandible spatulate to triangular, but basal angle always indistinct, masticatory margin with 1–4 teeth	**10**
10	Forewing with five closed cells, 1m–cu cross-vein present (Fig. 41A). In profile, petiolar peduncle longer than postpetiolar length (Fig. 41C)	***Syllophopsis***
–	Forewing with four closed cells, 1m–cu cross-vein absent (Fig. 41B). In profile, petiolar peduncle absent or shorter than postpetiolar length (Fig. 41D)	**11**
11	With head in full-face view, antennal scape short, barely reaching posterior ocular margin; mandible long and curved, masticatory margin with 3 or 4 teeth (Fig. 42A)	***Monomorium***
–	With head in full-face view, antennal scape long reaching occipital margin; mandible short and spatulate, basal margin linear, unidentate (Fig. 42B)	***Adelomyrmex*** (Seychelles)
12	Antennal scrobe runs below eyes (Fig. 43A)	***Cataulacus***
–	Antennal scrobe absent or runs above eyes (Fig. 43B)	**13**
13	Protibia without spur (Fig. 44A)	***Melissotarsus***
–	Protibia with single spur (Fig. 44B)	**14**
14	In lateral view, mesonotal suture extends downward from transverse suture to upper margin of mesopleuron, ending higher than highest point of wing insertion (Fig. 45A)	***Terataner***
–	In lateral view, mesonotal suture situated at same level or lower than highest point of wing insertion (Fig. 45B)	**15**
15	Abdominal segment III attached dorsally to abdominal segment IV (Fig. 46A). Scape and remaining segments same size (Fig. 46C)	***Crematogaster***
–	Abdominal segment III broadly attached to abdominal segment IV or abdominal segment III anteriorly attached to abdominal segment IV (Fig. 46B). Scape and remaining segments vary in size (Fig. 46D)	**16**
16	Peduncle of abdominal segment III distinctly longer than that of abdominal segment II (Fig. 47A)	***Eutetramorium***
–	Peduncle of abdominal segment III absent or shorter than that of abdominal segment II (Fig. 47B)	**17**
17	Second funicular segment distinctly more elongated than remaining segments, length nearly or more than twice as long as that of third funicular segment (Fig. 48A)	**18**
–	Second funicular segment not more elongated than remainder; even if it is elongated, length distinctly less than twice as long as that of third funicular segment (Fig. 48B)	**19**
18	Notauli present (Fig. 49A)	***Tetramorium***
–	Notauli absent (Fig. 49B)	***Dicroaspis*** (Comoros)
19	With head in full-face view, occipital carina visible (Fig. 50A)	***Malagidris***
–	With head in full-face view, occipital carina not visible (Fig. 50B)	**20**
20	Antennal scrobe clearly present (Fig. 51A)	***Metapone***
–	Antennal scrobe reduced to absent (Fig. 51B)	**21**
21	Antenna with 12 segments	**22**
–	Antenna with 13 segments	**23**
22	Cross-vein 1m-Cu present. Propodeum armed with a weakly developed angular tooth (Fig. 52A)	***Calyptomyrmex*** (Comoros)
–	Cross-vein 1m-Cu absent. Propodeum unarmed and round (Fig. 52B)	***Pristomyrmex*** (Mauritius)
23	Propodeal spines distinctly present (Fig. 53A)	***Cardiocondyla***
–	Propodeal spines absent (Fig. 53B)	**24**
24	Radial sector vein on forewing is curved toward costal margin distal to wing stigma and often reaches costal margin (Fig. 54A). Vertex is clearly divided from occiput by distinct occipital carina	**25**
–	Radial sector vein on forewing is downcurved and never reaches costal margin (Fig. 54B). Occipital carina is unclear or very weakly present, vertex slopes to occiput gently and gradually and is not divided by a carina	**27**
25	Abdominal segment III broadly attaches to abdominal segment IV (Fig. 55A)	***Carebara***
–	Abdominal segment III narrowly attaches to abdominal segment IV (Fig. 55B)	**26**
26	Mandible edentate (Fig. 56A)	***Meranoplus***
–	Mandible with 3–5 teeth which decrease in size from apex to base (Fig. 56B)	***Nesomyrmex***
27	Mandible edentate (Fig. 57A)	***Vollenhovia*** (Seychelles)
–	Mandible distinctly toothed (Fig. 57B)	**28**
28	Notauli absent (Fig. 58A)	***Trichomyrmex***
–	Notauli present (Fig. 58B)	**29**
29	Masticatory margin with 5–7 teeth (Fig. 59A), forewing with a dense fringe of long hairs along margin (Fig. 59C)	***Vitsika***
–	Masticatory margin with 2 or 3 teeth (Fig. 59B), forewing lacking long hairs on edges (Fig. 59D)	***Royidris***

**Figure 33.**
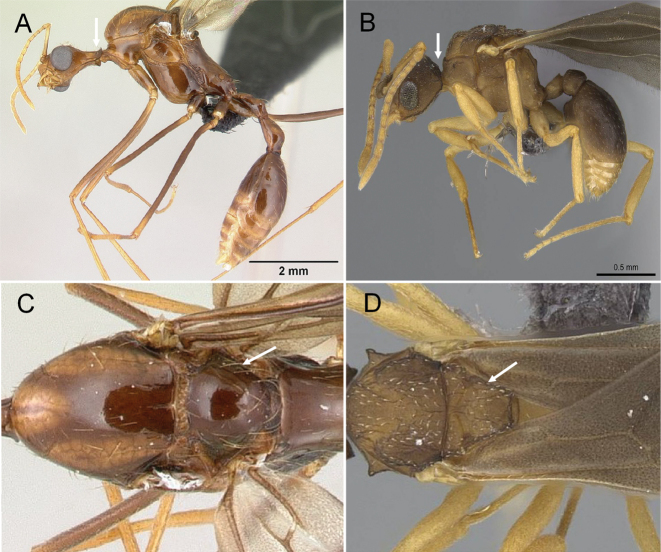
In profile view showing occipital carina **A, C***Aphaenogasterbressleri* (CASENT0495103). In dorsal view showing mesoscutellum **B, D***Cyphomyrmexminutus* (CASENT0264488). Photographers April Nobile (**A, C**), Michele Esposito (**B, D**).

**Figure 34.**
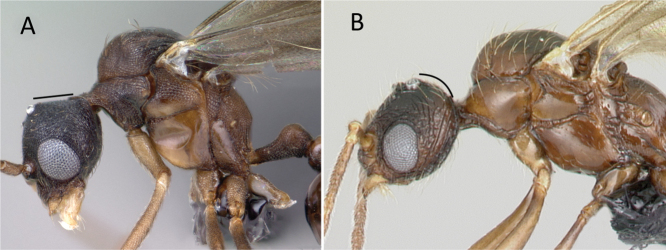
Head in profile view **A***Strumigenyschilo* (CASENT0145240) **B***Tetramoriumsilvicola* (CASENT0494732). Photographers Dimby Raharinjanahary (**A**), Erin Prado (**B**).

**Figure 35.**
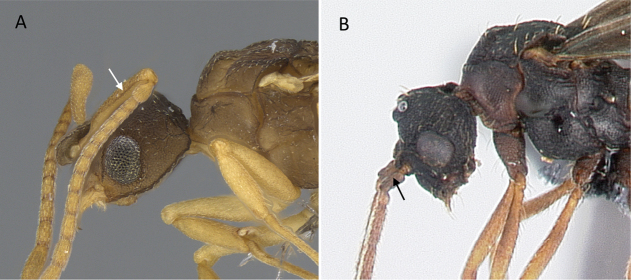
Scape length in profile view **A***Cyphomyrmexminutus* (CASENT0264488) **B***Eurhopalothrix* km01 (CASENT0146071). Photographers Michele Esposito (**A**), Erin Prado (**B**).

**Figure 36.**
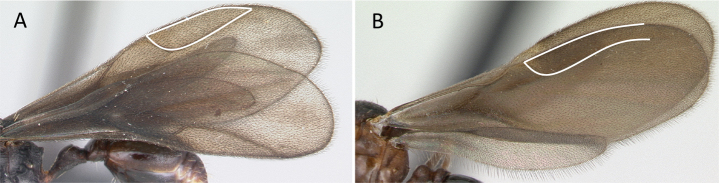
Forewing in lateral view showing radial sector vein **A***Eurhopalothrix* km01 (CASENT0146071) **B***Strumigenysdicomas* (CASENT0135118). Photographer Erin Prado.

**Figure 37.**
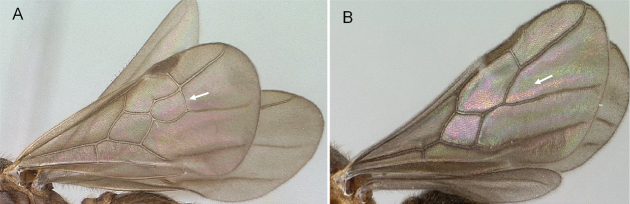
Forewing in lateral view showing cross vein 2rs-m **A***Pheidole* mgs006 (CASENT0135889) **B***Carebara* drm03 (CASENT0143975). Photographer Dimby Raharinjanahary.

**Figure 38.**
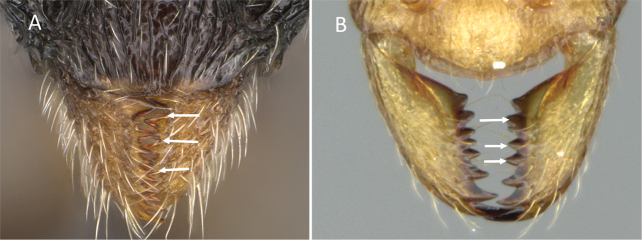
Mandible in full-face view **A***Pilotrochusbesmerus* (CASENT0083498) **B***Malagidrissofina* (CASENT0906626). Photographers Michele Esposito (**A**), Estella Ortega (**B**).

**Figure 39.**
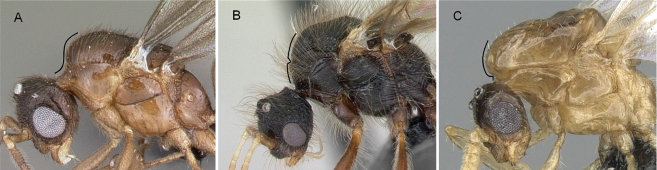
Head and mesosoma in profile view **A***Monomoriumtermitobium* (CASENT0460162) **B***Meranoplusmayri* (CASENT0062813) **C***Crematogasterhazolava* (CASENT0317643). Photographers April Nobile (**A, B**), Estella Ortega (**C**).

**Figure 40.**
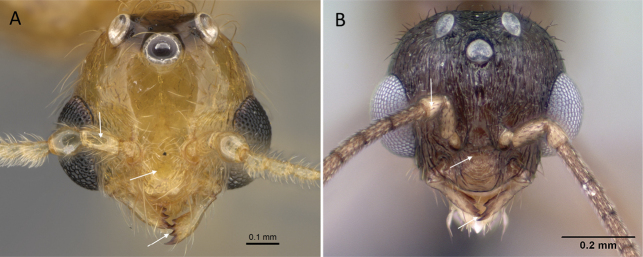
Head in full-face view showing first funicular segment, mandible, and postero-median margin of clypeus **A***Erromyrmalatinodis* (CASENT0788835) **B***Syllophopsismodesta* (CASENT0143818). Photographers Michele Esposito (**A**), Dimby Raharinjanahary (**B**).

**Figure 41.**
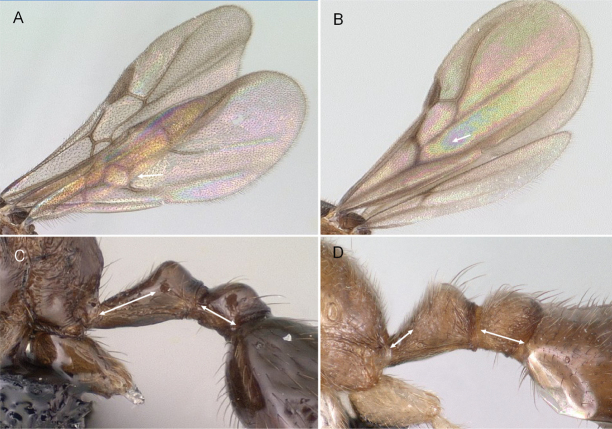
Forewing. Abdominal segment II and abdominal segment III in lateral view showing 1m–cu cross-vein and peduncular length **A, C***Syllophopsismodesta* (CASENT0135642) **B***Monomoriumtermitobium* (CASENT0135673) **D***Monomoriumtermitobium* (CASENT0135952). Photographer Dimby Raharinjanahary.

**Figure 42.**
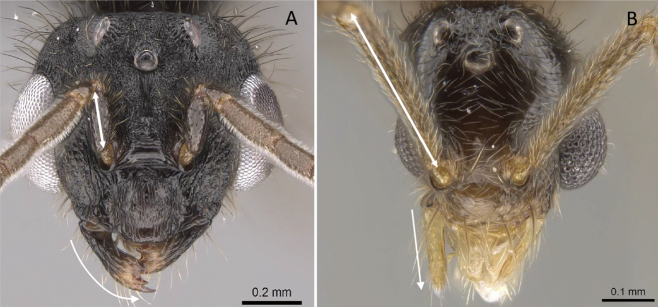
Head in full-face view showing form of mandible and scape length **A***Monomoriummadecassum* (CASENT0209350) **B***Adelomyrmex* sc01 (CASENT0160764). Photographer Michele Esposito.

**Figure 43.**
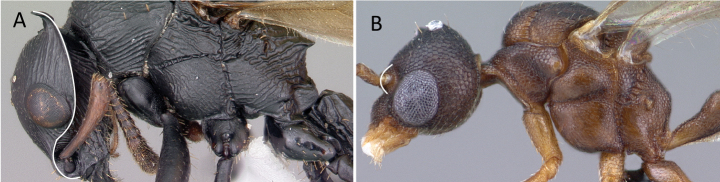
Head in lateral view showing position of antennal scrobe **A***Cataulacusoberthueri* (CASENT0435930) **B***Strumigenysambatrix* (CASENT0135807). Photographers April Nobile (**A**), Dimby Raharinjanahary (**B**).

**Figure 44.**
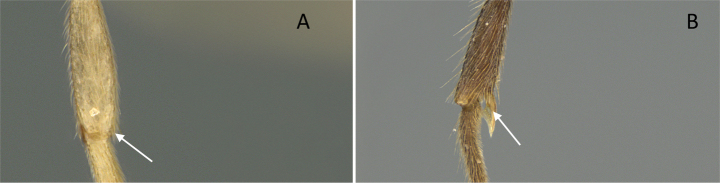
Protibia in ventral view **A***Melissotarsusinsularis* (CASENT0804569) **B***Terataner* fhg22 (CASENT0429745). Photographer Michele Esposito.

**Figure 45.**
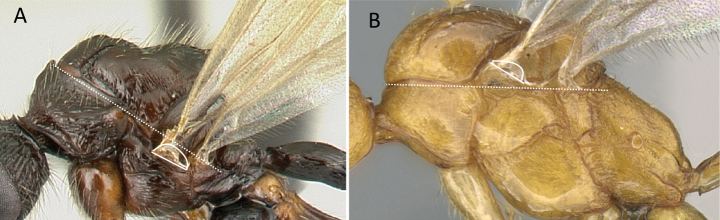
Mesosoma in lateral view showing position of mesonotal suture relative to point of wing process **A***Terataneralluaudi* (CASENT0496102) **B***Malagidrisdulcis* (CASENT0135071). Photographers Erin Prado (**A**), Estella Ortega (**B**).

**Figure 46.**
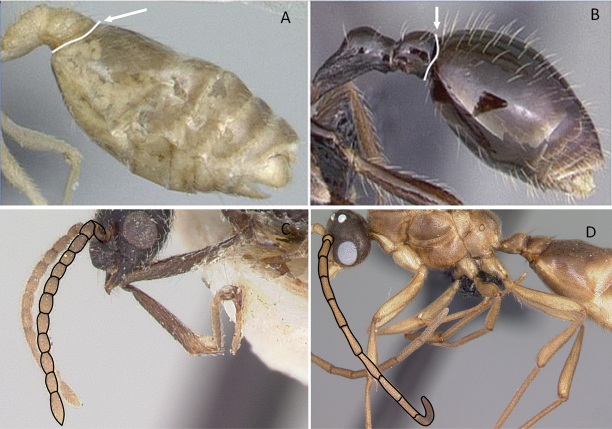
Abdominal segment III attachment to abdominal segment IV **A***Crematogastermaina* (CASENT0132785) **B***Pilotrochusbesmerus* (CASENT0083498). Size comparison of scape and remaining segments **C***Crematogasteragnetis* (CASENT0101760) **D***Carebarajajoby* (CASENT0494540). Photographers Estella Ortega (**A**), April Nobile (**B–D**)

**Figure 47.**
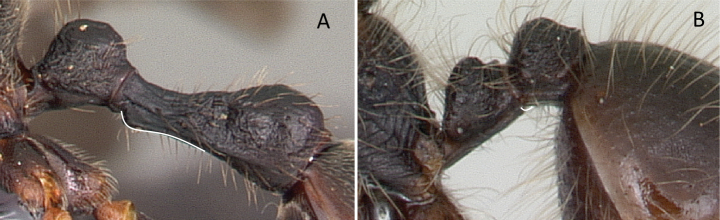
Abdominal segment II and III in lateral view showing the peduncular length **A***Eutetramoriummocquerysi* (CASENT0495192) **B***Meranoplusmayri* (CASENT0062813). Photographer April Nobile.

**Figure 48.**
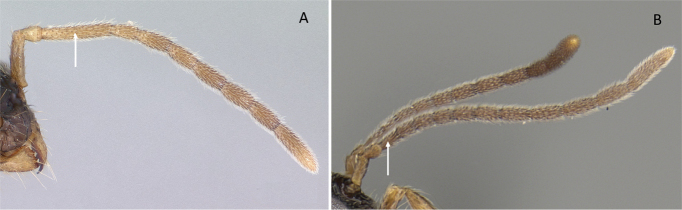
Antennae in lateral view showing the length of second funicular segment **A***Tetramoriummars* (CASENT0134555) **B***Pilotrochusbesmerus* (CASENT0057183). Photographers Dimby Raharinjanahary (**A**), Michele Esposito (**B**).

**Figure 49.**
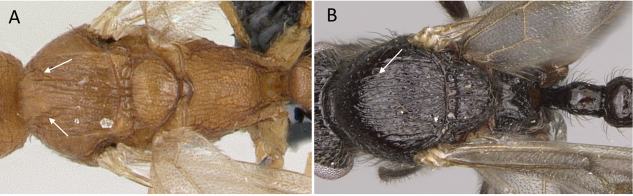
Promesonotum in dorsal view **A***Tetramoriumkelleri* (CASENT0133425) **B***Dicroaspis* sp. (CASENT0389458). Photographers Erin Prado (**A**), Michele Esposito (**B**).

**Figure 50.**
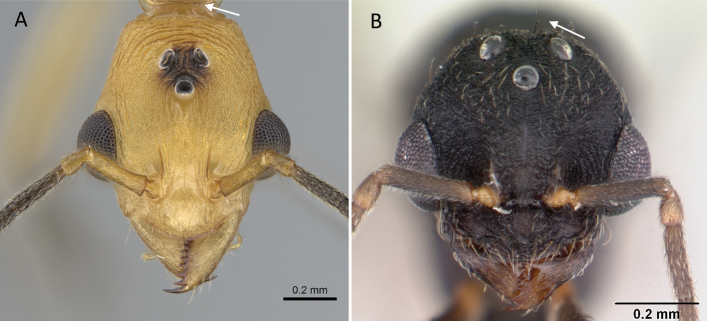
Head in full-face view showing occipital carina **A***Malagidrisalperti* (CASENT0248385) **B***Calyptomyrmex* km01 (CASENT0136409). Photographers Michele Esposito (**A**), April Nobile (**B**).

**Figure 51.**
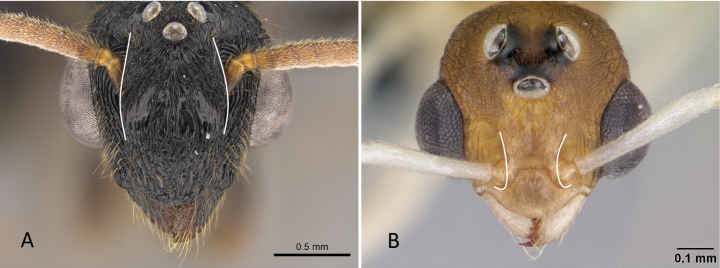
Head in full-face view showing antennal scrobe **A***Metaponeemersoni* (CASENT0113799) **B***Nesomyrmexangulatus* (CASENT0147245). Photographers Michele Esposito (**A**), Erin Prado (**B**).

**Figure 52.**
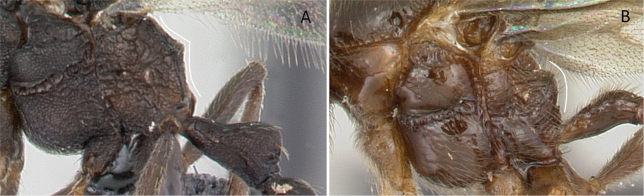
Propodeum in lateral view **A***Calyptomyrmex* km01 (CASENT0136409) **B***Pristomyrmexbispinosus* (CASENT0055726). Photographer April Nobile.

**Figure 53.**
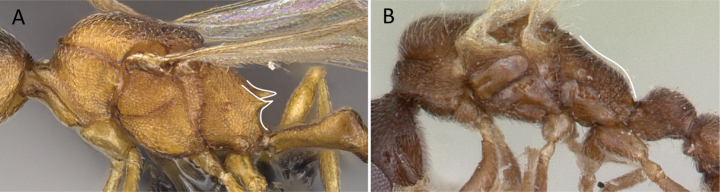
Propodeal spines in lateral view **A***Cardiocondylaemeryi* (CASENT0082706) **B***Vollenhoviapiroskae* (CASENT0101658). Photographers Michele Esposito (**A**), April Nobile (**B**).

**Figure 54.**
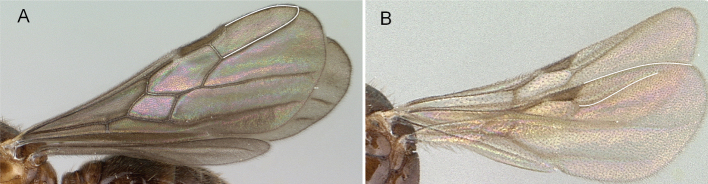
Forewing showing Rs reaching the costal margin **A***Carebara* drm03 (CASENT0143975) **B***Monomoriumexiguum* (CASENT0135614). Photographer Dimby Raharinjanahary.

**Figure 55.**
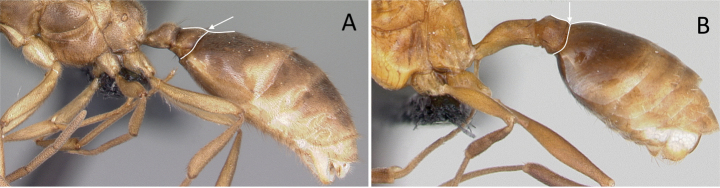
Abdomen in lateral view showing the attachment of abdominal segment III **A***Carebarajajoby* (CASENT0494540) **B***Nesomyrmexhafahafa* (CASENT0053313). Photographer April Nobile.

**Figure 56.**
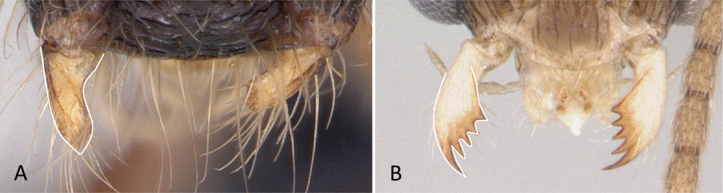
Mandible in full-face view **A***Meranoplusmayri* (CASENT0062813) **B***Nesomyrmextamatavensis* (CASENT0496295). Photographers April Nobile (**A**), Erin Prado (**B**).

**Figure 57.**
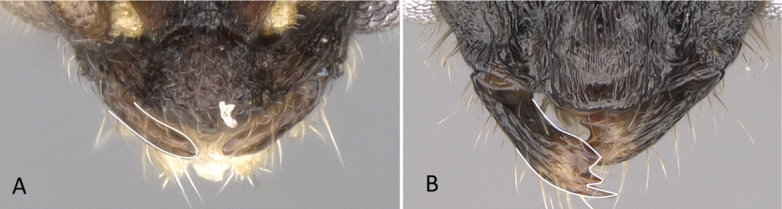
Mandible in full-face view **A***Vollenhoviapiroskae* (CASENT0159914) **B***Monomoriummadecassum* (CASENT0209350). Photographer Michele Esposito.

**Figure 58.**
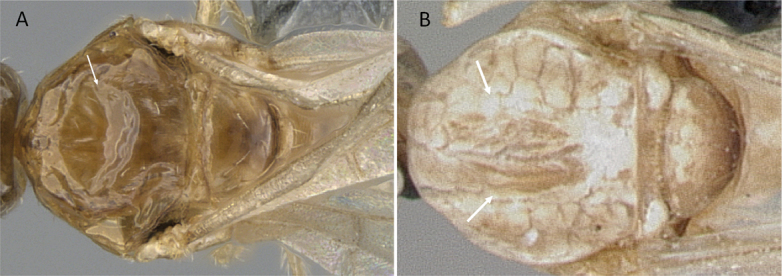
Promesonotum in dorsal view **A***Trichomyrmexdestructor* (CASENT0787666) **B***Royidrisnotorthotenes* (CASENT0002249) Photographers Michele Esposito (**A**). April Nobile (**B**).

**Figure 59.**
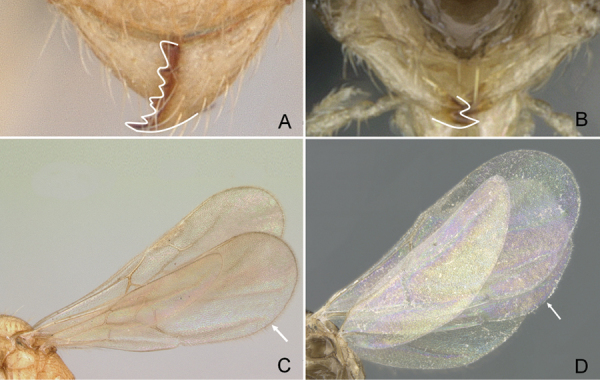
Mandible in full-face view and forewing fringe features in profile view **A, C***Vitsikacrebra* (CASENT0050262) **B, D***Royidrisperegrina* (CASENT0206165). Photographers April Nobile (**A, C**), Estella Ortega (**B, D**).

#### *Adelomyrmex* Emery, 1897

Mandible edentate. Palp formula unknown. Antennal scrobe absent. Antenna with 13 segments. First funicular segment not globular, shorter than scape. Scape very long, extending to margin of head. Length of first funicular segment is equal to first flagellar segment. In full-face view, eye located above of base of clypeus. Ocelli placed well below occipital margin in front view. Occipital carina invisible. With head full-face view, width excluding eyes is not distinctly narrowed anteriorly from level of posterior margin of eyes: width at level of posterior edge of eyes is not twice as wide as that at level of mandibular insertions. Mesoscutum in profile strongly overhangs pronotum, latter not visible in dorsal view. Notauli absent. With mesopleuron in lateral view, anterodorsal portion lower than highest point of wing process. Protibia with pectinate tibial spur. Mesotibia tibial spur absent. Metatibia tibial spur absent. Aroliae small. Propodeum unarmed and round. Abdominal segment II with a long anterior peduncle. Abdominal segment III narrowly attaches to abdominal segment IV. Paramere small. Pygostyle absent. Pubescence short, dense over most of body. On forewing, pterostigma reduced in size. Costal vein (C) present. Media between Rs+M and 2r-rs completely absent. Media (M) never reaching costal margin. Radial sector vein (Rs) never reaching costal margin. Cross-vein 2r-rs connected with radial sector vein posterior to pterostigma. Cross-vein 2rs-m absent. Cross-vein 1m-cu absent. Rs+M absent. Radius vein (R) absent. Cross-vein cu-a proximal to junction between media and cubitus. Cu absent. Free section of cubitus absent.

#### *Aphaenogaster* Mayr, 1853

Mandible with 3–6 teeth which decrease in size from apex to base. Palp formula 3,2. Antennal scrobe absent. Antenna with 13 segments. First funicular segment not globular, shorter than scape. Scape not short, reaching lower edge of margin of lateral ocelli. Eyes large, at or in front of midlength of sides. Ocelli placed well below occipital margin in front view. Occipital carina strongly developed, forming a nuchal collar. With head full-face view, width excluding eyes is not distinctly narrowed anteriorly from level of posterior margin of eyes: width at level of posterior edge of eyes is not twice as wide as that at level of mandibular insertions. Mesoscutum in profile strongly overhangs pronotum, latter not visible in dorsal view. Notauli present. With mesopleuron in lateral view, anterodorsal portion lower than highest point of wing process. Protibia with pectinate tibial spur. Mesotibia tibial spur absent. Metatibia tibial spur simple. Aroliae small. Propodeum unarmed, sometimes with short teeth/denticles. Abdominal segment II with a long anterior peduncle, spiracle located at apex of peduncle. Abdominal segment III narrowly attaches to abdominal segment IV. Paramere large. Pygostyle present. Pilosity simple throughout body. On forewing, pterostigma well developed. Costal vein (C) present. Media (M) fused with Rs+M. Media (M) never reaching costal margin. Radial sector vein (Rs) never reaching costal margin. Cross-vein 2r-rs connected with radial sector vein posterior to pterostigma. Cross-vein 2rs-m absent. Cross-vein 1m-cu present. Fusion of Rs+M extended distally so that 1m-cu arises from Rs+M not from M. Radius vein (R) present. Cross-vein cu-a proximal to junction between media and cubitus. Cu present. Free section of cubitus present.

#### *Calyptomyrmex* Emery, 1887

Mandible triangular and distinctly dentate, with five or six teeth which decrease in size from apex to base. Palp formula 2,2. Antennal scrobe reduced. Antenna with 12 segments. First funicular segment not globular, shorter than scape. Scape short, not reaching lower edge of margin of lateral ocelli. Eyes large, at or in front of midlength of sides. Ocelli placed near occipital margin in front view. Occipital carina invisible. With in head full-face view, width excluding eyes is not distinctly narrowed anteriorly from level of posterior margin of eyes: width at level of posterior edge of eyes is not twice as wide as that at level of mandibular insertions. Mesoscutum punctate. Notauli absent. With mesopleuron in lateral view, anterodorsal portion lower than highest point of wing process. Protibia with pectinate tibial spur. Mesotibia tibial spur absent. Metatibia tibial spur absent. Aroliae small. Propodeum armed, projects at a low angle. Abdominal segment II with a long anterior peduncle, spiracle located at apex of peduncle. Abdominal segment III narrowly attaches to abdominal segment IV. Paramere small. Pygostyle absent. Pilosity simple throughout body. On forewing, pterostigma well developed. Costal vein (C) present. Media (M) fused with Rs+M. Media (M) never reaching costal margin. Radial sector vein (Rs) never reaching costal margin. Cross-vein 2r-rs connected with radial sector vein posterior to pterostigma. Cross-vein 2rs-m absent. Cross-vein 1m-cu present. Fusion of Rs+M extended distally, so that 1m-cu arises from Rs+M, not from M. Radius vein (R) absent. Cross-vein cu-a proximal to junction between media and cubitus. Cu present. Free section of cubitus absent.

Remarks. The Malagasy species, *Calyptomyrmex* km01 does not have notauli, in contrast to the descriptions by Ito et al. (2023) and Emery (1922).

#### *Cardiocondyla* Emery, 1869

Ergatoid males of *Cardiocondyla* are easily distinguished by having long, toothless, and saber-shaped mandibles for *Cardiocondylawroughtonii* but worker-like mandibles have been observed in *Cardiocondylaemeryi* and *Cardiocondylashuckardi*, and reduced black pigmentation (leading to a pale yellowish-brown overall coloration), decreased eye size, and partially or completely reduced ocelli (Seifert 2003).

In winged males, mandible reduced, short, and narrow, with only five teeth. Palp formula 2,2. Antennal scrobe reduced. Antenna with 13 segments. First funicular segment not globular, shorter than scape. Scape short, not reaching lower edge of margin of lateral ocelli. In full-face view, eye located above base of clypeus. Ocelli placed well below occipital margin in front view. Occipital carina invisible. With head in full-face view, width excluding eyes is not distinctly narrowed anteriorly from level of posterior margin of eyes: width at level of posterior edge of eyes is not twice as wide as that at level of mandibular insertions. Mesoscutum punctate. Notauli absent. With mesopleuron in lateral view, anterodorsal portion lower than highest point of wing process. Protibia with pectinate tibial spur. Mesotibia tibial spur absent. Metatibia tibial spur absent. Aroliae small. Propodeum armed. Abdominal segment II with a long anterior peduncle. Abdominal segment III narrowly attaches to abdominal segment IV. Paramere small. Pygostyle absent. Pubescence short, dense over most of body. On forewing, pterostigma reduced in size. Costal vein (C) absent. Media between Rs+M and 2r-rs completely absent. Media (M) never reaching costal margin. Radial sector vein (Rs) never reaching costal margin. Cross-vein 2r-rs connected with radial sector vein posterior to pterostigma. Cross-vein 2rs-m absent. Cross-vein 1m-cu absent. Rs+M absent. Radius vein (R) absent. Cross-vein cu-a absent. Cu absent. Free section of cubitus absent.

#### *Carebara* Westwood, 1840

Mandible reduced, with three or four teeth which decrease in size from apex to base. Palp formula 3,2. Antennal scrobe absent. Antenna with 13 segments. First funicular segment not globular, shorter than scape. Scape shorter than second funicular segment. Eyes large, at or in front of midlength of sides. Ocelli placed near occipital margin in front view. Occipital carina invisible. With head in full-face view, width excluding eyes is not distinctly narrowed anteriorly from level of posterior margin of eyes: width at level of posterior edge of eyes is not twice as wide as that at level of mandibular insertions. Mesoscutum in profile strongly overhangs pronotum, latter not visible in dorsal view. Notauli absent with a longitudinal median carina that is narrowly bifurcated anteriorly. With mesopleuron in lateral view, anterodorsal portion lower than highest point of wing process. Protibia with pectinate tibial spur. Mesotibia tibial spur absent. Metatibia tibial spur absent. Aroliae small. Propodeum unarmed and round. Abdominal segment II with a short, stout anterior peduncle and a short but relatively high node. Abdominal segment III broadly attaches to abdominal segment IV. Paramere large. Pygostyle present. Pubescence short, dense over most of body. On forewing, pterostigma well developed. Costal vein (C) present. Media (M) fused with Rs+M. Media (M) reaches costal margin. Radial sector vein (Rs) reaches costal margin. Cross-vein 2r-rs connected with radial sector vein posterior to pterostigma. Cross-vein 2rs-m absent. Cross-vein 1m-cu present. Fusion of Rs+M extended distally, so that 1m-cu arises from Rs+M, not from M. Radius vein (R) present. Cross-vein cu-a proximal to junction between media and cubitus. Cu present. Free section of cubitus present.

#### *Cataulacus* Smith, 1853

Mandible triangular with denticles which decrease in size from apex to base. Palp formula 4,2. Antennal scrobe running below eyes. Antenna with 11 segments (Emery 1922; Bolton 1974). Length of first funicular is equal to that of second funicular segment + third funicular segment. Scape short, not reaching lower edge of margin of lateral ocelli. In full-face view, eye located in front of midlength of head capsule. Ocelli placed well below occipital margin in front view. Occipital carina invisible. With head in full-face view, width excluding eyes is not distinctly narrowed anteriorly from level of posterior margin of eyes: width at level of posterior edge of eyes is not twice as wide as that at level of mandibular insertions. Mesoscutum striate. Notauli present. With mesopleuron in lateral view, anterodorsal portion lower than highest point of wing process. Protibia with pectinate tibial spur. Mesotibia tibial spur absent. Metatibia tibial spur absent. Aroliae small. Propodeum unarmed. Abdominal segment II without a long anterior peduncle. Abdominal segment III narrowly attaches to abdominal segment IV. Paramere visible. Pygostyle absent. Pilosity simple throughout body. On forewing, pterostigma reduced in size. Costal vein (C) absent. Media between Rs+M and 2r-rs completely absent. Media (M) never reaching costal margin. Radial sector vein (Rs) never reaching costal margin. Cross-vein 2r-rs connected with radial sector vein posterior to pterostigma. Cross-vein 2rs-m absent. Cross-vein 1m-cu absent. Rs+M merge with Rs. Radius vein (R) absent. Cross-vein cu-a absent. Cu absent. Free section of cubitus absent.

#### *Crematogaster* Lund, 1831

Mandible triangular edentate or dentate with one or two teeth. Palp formula 3,2; 5,3. Antennal scrobe is absent. Antenna with 11 or 12 segments. First funicular segment subglobular, shorter than scape. Scape shorter than 1+2 flagellar segment. Eyes large, at or in front of midlength of sides. Ocelli placed near occipital margin in front view. Occipital carina invisible. With head in full-face view, width excluding eyes is not distinctly narrowed anteriorly from level of posterior margin of eyes: width at level of posterior edge of eyes is not twice as wide as that at level of mandibular insertions. Mesoscutum in profile strongly overhangs pronotum, latter not visible in dorsal view. Notauli absent. With mesopleuron in lateral view, anterodorsal portion lower than highest point of wing process. Protibia with pectinate tibial spur. Mesotibia tibial spur absent. Metatibia tibial spur absent. Aroliae small. Propodeum unarmed and round. Abdominal segment II and Abdominal segment III are equal in size. Abdominal segment III dorsally attaches to abdominal segment IV. Paramere large. Pygostyle present. Pilosity simple throughout body. On forewing, pterostigma well developed. Costal vein (C) present. Media (M) between Rs+M and 2rs-m and after 2rs-m completely present. Media (M) never reaching costal margin. Radial sector vein (Rs) never reaching costal margin. Cross-vein 2r-rs connected with radial sector vein posterior to pterostigma. Cross-vein 2rs-m absent. Cross-vein 1m-cu present. Rs+M present. Radius vein (R) present. Cross-vein cu-a proximal to junction between media and cubitus. Cu present. Free section of cubitus absent.

#### *Cyphomyrmex* Mayr, 1862

Mandible triangular with three teeth. Palp formula 2,2. Antennal scrobe running above eyes. Antenna with 13 segments. First funicular segment not globular, shorter than scape. Eyes large, at or in front of midlength of sides Ocelli placed near occipital margin in front view. Occipital carina invisible. With head in full-face view, width excluding eyes is distinctly narrowed anteriorly from level of posterior margin of eyes: width at level of posterior margin of eyes is nearly twice as wide as that at level of mandible insertions. Pronotum anterodorsally sharply marginate, with sharp, dentate corners. Notauli present. With mesopleuron in lateral view, anterodorsal portion lower than highest point of wing process. Protibia pectinate tibial spur. Mesotibia tibial spur absent. Metatibia tibial spur absent. Aroliae small. Propodeum armed or angle projects as a low, obtuse tooth. Abdominal segment II with a short peduncle. Abdominal segment III narrowly attaches to abdominal segment IV. Paramere visible. Pygostyle present. Pilosity simple throughout body. On forewing, pterostigma reduced in size. Costal vein (C) present. Media between Rs+M and 2r-rs completely absent. Media (M) never reaching costal margin. Radial sector vein (Rs) reaches costal margin. Cross-vein 2r-rs connected with radial sector vein posterior to pterostigma. Cross-vein 2rs-m absent. Cross-vein 1m-cu absent. Rs+M merge with Rs. Radius vein (R) present. Cross-vein cu-a absent. Cu absent. Free section of cubitus absent.

#### *Dicroaspis* Emery, 1908

Mandible triangular with seven teeth. Antennal scrobe running above eyes. Antenna with ten segments. First funicular segment not globular, shorter than scape. Scape very long, extending to margin of head. Eyes large, at or in front of midlength of sides. Ocelli placed well below occipital margin in front view. Occipital carina invisible. With head full-face view, width excluding eyes is not distinctly narrowed anteriorly from level of posterior margin of eyes: width at level of posterior edge of eyes is not twice as wide as that at level of mandibular insertions. Pronotum anterodorsally sharply marginate, with sharp, dentate corners. Notauli absent. With mesopleuron in lateral view, anterodorsal portion lower than highest point of wing process. Protibia with pectinate tibial spur. Mesotibia tibial spur absent. Metatibia tibial spur absent. Aroliae small. Propodeum unarmed and round. Abdominal segment II with a long peduncle. Abdominal segment III narrowly attaches to abdominal segment IV. Paramere visible. Pygostyle present. Pilosity simple throughout body. On forewing, pterostigma well developed. Costal vein (C) present. Media (M) fused with Rs+M. Media (M) never reaching costal margin. Radial sector vein (Rs) reaches costal margin. Cross-vein 2r-rs connected with radial sector vein posterior to pterostigma. Cross-vein 2rs-m absent. Cross-vein 1m-cu absent. Rs+M merge with Rs. Radius vein (R) absent. Cross-vein cu-a proximal to junction between media and cubitus. Cu absent. Free section of cubitus absent.

#### *Erromyrma* Bolton & Fisher, 2016

Mandible triangular (Fisher and Bolton 2016; Ramamonjisoa et al. 2023), short, and narrow, with only four or five teeth. Palp formula 5,3. Antennal scrobe absent. Antenna with 13 segments. First funicular segment subglobular, same size as scape. Eyes large, at or in front of midlength of sides. Ocelli placed close to occipital margin in front view. Occipital carina invisible. With head in full-face view, width excluding eyes is not distinctly narrowed anteriorly from level of posterior margin of eyes: width at level of posterior edge of eyes is not twice as wide as that at level of mandibular insertions. Mesoscutum in profile strongly overhangs pronotum, latter not visible in dorsal view. Notauli absent. With mesopleuron in lateral view, anterodorsal portion lower than highest point of wing process. Protibia with pectinate tibial spur. Mesotibia tibial spur absent. Metatibia tibial spur absent. Aroliae small. Propodeum unarmed and round. Abdominal segment II with a short peduncle. Abdominal segment III narrowly attaches to abdominal segment IV. Paramere visible. Pygostyle present. Pilosity simple throughout body. On forewing, pterostigma well developed. Costal vein (C) absent. Media (M) fused with Rs+M. Media (M) never reaching costal margin. Radial sector vein (Rs) never reaching costal margin. Cross-vein 2r-rs connected with radial sector vein posterior to pterostigma. Cross-vein 2rs-m absent. Cross-vein 1m-cu present. Fusion of Rs+M extended distally, so that 1m-cu arises from Rs+M, not from M. Radius vein (R) present. Cross-vein cu-a proximal to junction between media and cubitus. Cu present. Free section of cubitus present.

#### *Eurhopalothrix* Brown & Kempf, 1961

Mandible triangular edentate. Palp formula 2,2. Antennal scrobe running above eyes. Antenna with 13 segments. First funicular segment not globular, shorter than scape. Eyes large, at or in front of midlength of sides. Ocelli placed near occipital margin in front view. Occipital carina invisible. With head in full-face view, width excluding eyes is distinctly narrowed anteriorly from level of posterior margin of eyes: width at level of posterior margin of eyes is nearly twice as wide as that at level of mandible insertions. Mesoscutum punctate. Notauli absent. With mesopleuron in lateral view, anterodorsal portion lower than highest point of wing process. Protibia with pectinate tibial spur. Mesotibia tibial spur absent. Metatibia tibial spur absent. Aroliae small. Propodeum angle projects as a low, obtuse tooth. Abdominal segment II with a long anterior peduncle. Abdominal segment III narrowly attaches to abdominal segment IV. Paramere small. Pygostyle present. Pilosity simple throughout body. On forewing, pterostigma reduced in size. Costal vein (C) absent. Media between Rs+M and 2r-rs completely absent. Media (M) never reaching costal margin. Radial sector vein (Rs) reaches costal margin. Cross-vein 2r-rs connected with radial sector vein posterior to pterostigma. Cross-vein 2rs-m absent. Cross-vein 1m-cu absent. Rs+M merge with Rs. Radius vein (R) present. Cross-vein cu-a absent. Cu absent. Free section of cubitus absent.

#### *Eutetramorium* Emery, 1899

Mandible stoutly triangular, with seven teeth. Palp formula 4,3. Antennal scrobe is absent. Antenna with 13 segments. SI 31. First funicular segment long but not globular, ~ 25% longer than length of second funicular segment. In full-face view, eye located in front of midlength of head capsule. Ocelli placed well below occipital margin in front view. Occipital carina sharp but not forming a raised crest. With head in full-face view, width excluding eyes is not distinctly narrowed anteriorly from level of posterior margin of eyes: width at level of posterior edge of eyes is not twice as wide as that at level of mandibular insertions. Anterior mesoscutum between notauli arms, with a longitudinal median carina that is narrowly bifurcated anteriorly. Notauli weakly present, anterior arms forming a V-shape. With mesopleuron in lateral view, anterodorsal portion lower than highest point of wing process. Protibia with pectinate tibial spur. Mesotibia tibial spur simple. Metatibia tibial spur simple. Aroliae small. Propodeum unarmed, spiracle low on side and in front of midlength of sclerite; propodeal lobes conspicuous, rounded. Abdominal segment II with a short, stout anterior peduncle and a short but relatively high node, spiracle approx. level with base of anterior face of node. Abdominal segment III greatly elongated, in profile almost twice length of abdominal segment II. Abdominal segment III narrowly attaches to abdominal segment IV. Paramere small. Pygostyle present. Denser upright pilosity. On forewing, pterostigma reduced in size. Costal vein (C) absent. Media (M) fused with Rs+M. Media (M) never reaching costal margin. Radial sector vein (Rs) never reaching costal margin. Cross-vein 2r-rs connected with radial sector vein posterior to pterostigma. Cross-vein 2rs-m absent. Cross-vein 1m-cu present. Fusion of Rs+M extended distally, so that 1m-cu arises from Rs+M, not from M. Radius vein (R) absent. Cross-vein cu-a proximal to junction between media and cubitus. Cu present. Free section of cubitus present.

#### *Malagidris* Bolton & Fisher, 2014

Mandible triangular and strongly dentate, with nine sharp teeth. Palp formula 3,2. Antennal scrobe is reduced. Antenna with 13 segments. First funicular segment short, not globular, ~ ¼–1/2 length of second funicular segment. In full-face view, eye located in front of midlength of head capsule. Ocelli placed near occipital margin in front view. Occipital carina sharp, forming a distinct crest. With head in full-face view, width excluding eyes is not distinctly narrowed anteriorly from level of posterior margin of eyes: width at level of posterior edge of eyes is not twice as wide as that at level of mandibular insertions. Mesoscutum convex in profile, mesoscutum and mesoscutellum elevated, much higher than propodeal dorsum, which is depressed and slopes downward posteriorly. Notauli absent. With mesopleuron in lateral view, anterodorsal portion lower than highest point of wing process. Protibia with pectinate tibial spur. Mesotibia tibial spur simple. Metatibia tibial spur simple. Aroliae small. Propodeum unarmed, spiracle high on side and at approx. midlength, or slightly in front of midlength, of sclerite; propodeal lobes conspicuous, rounded. Abdominal segment II with a long anterior peduncle and a low node, spiracle at or behind midlength of peduncle, but in front of level of node. Abdominal segment III narrowly attaches to abdominal segment IV. Paramere large. Pygostyle present. Pilosity scarce. On forewing, pterostigma well developed. Costal vein (C) present. Media (M) fused with Rs+M. Media (M) never reaching costal margin. Radial sector vein (Rs) reaches costal margin. Cross-vein 2r-rs connected with radial sector vein posterior to pterostigma. Cross-vein 2rs-m absent. Cross-vein 1m-cu present. Fusion of Rs+M extended distally, so that 1m-cu arises from Rs+M, not from M. Radius vein (R) present. Cross-vein cu-a proximal to junction between media and cubitus. Cu present. Free section of cubitus present.

#### *Melissotarsus* Emery, 1877

Mandible triangular edentate or dentate with one or two teeth. Palp formula 0,1. Antennal scrobe is reduced. Antenna with 12 segments. First funicular segment short, not globular, ~ 1/2 length of second funicular segment. In full-face view, eye located in front of midlength of head capsule. Ocelli placed close to occipital margin in front view. Occipital carina invisible. With head in full-face view, width excluding eyes is not distinctly narrowed anteriorly from level of posterior margin of eyes: width at level of posterior edge of eyes is not twice as wide as that at level of mandibular insertions. Mesoscutum convex in profile, mesoscutum and mesoscutellum elevated, much higher than propodeal dorsum, which is depressed and slopes downward posteriorly. Notauli absent. With mesopleuron in lateral view, anterodorsal portion lower than highest point of wing process. Protibia without tibial spur. Mesotibia tibial spur simple. Metatibia tibial spur simple. Aroliae small. Propodeum unarmed and round. Abdominal segment II without a long anterior peduncle. Abdominal segment III narrowly attaches to abdominal segment IV. Paramere large. Pygostyle absent. Pilosity simple throughout body. On forewing, pterostigma reduced in size. Costal vein (C) absent. Media (M) fused with Rs+M. Media (M) present. Radial sector vein (Rs) reaches costal margin. Cross-vein 2r-rs connected with radial sector vein posterior to pterostigma. Cross-vein 2rs-m absent. Cross-vein 1m-cu absent. Rs+M present. Radius vein (R) present. Cross-vein cu-a present. Cu absent. Free section of cubitus absent.

#### *Meranoplus* Smith, 1853

Mandible reduced, short, and narrow, with only one tooth. Palp formula 5,3. Antennal scrobe absent. Antenna with 13 segments. First funicular segment short, not globular, ~ 1/2 length of second funicular segment. In full-face view, eye located in front of midlength of head capsule. Ocelli placed near occipital margin in front view. Occipital carina invisible. With head in full-face view, width excluding eyes is not distinctly narrowed anteriorly from level of posterior margin of eyes: width at level of posterior edge of eyes is not twice as wide as that at level of mandibular insertions. Mesoscutum in profile strongly overhangs pronotum, latter not visible in dorsal view. Notauli present. With mesopleuron in lateral view, anterodorsal portion lower than highest point of wing process. Protibia with pectinate tibial spur. Mesotibia tibial spur simple. Metatibia tibial spur simple. Aroliae small. Propodeum unarmed and round. Abdominal segment II without a long anterior peduncle. Abdominal segment III narrowly attaches to abdominal segment IV. Paramere visible. Pygostyle absent. Pilosity long throughout body. On forewing, pterostigma well developed. Costal vein (C) present. Media (M) fused with Rs+M. Media (M) never reaching costal margin. Radial sector vein (Rs) reaches costal margin. Cross-vein 2r-rs connected with radial sector vein posterior to pterostigma. Cross-vein 2rs-m absent. Cross-vein 1m-cu present. Fusion of Rs+M extended distally, so that 1m-cu arises from Rs+M, not from M. Radius vein (R) present. Cross-vein cu-a proximal to junction between media and cubitus. Cu absent. Free section of cubitus absent.

#### *Metapone* Forel, 1911

Mandible triangular and distinctly dentate with four teeth. Palp formula 1,2. Antennal scrobe running above eyes. Antenna with 12 segments. First funicular segment short, not globular, about same size as second funicular segment. In full-face view, eye located in front of midlength of head capsule. Ocelli placed well below occipital margin in front view. Occipital carina invisible. With head in full-face view, width excluding eyes is not distinctly narrowed anteriorly from level of posterior margin of eyes: width at level of posterior edge of eyes is not twice as wide as that at level of mandibular insertions. Mesoscutum striate. Notauli absent. With mesopleuron in lateral view, anterodorsal portion lower than highest point of wing process. Protibia with pectinate tibial spur. Mesotibia tibial spur absent. Metatibia tibial spur simple. Aroliae small. Propodeum unarmed. Abdominal segment II without peduncle. In profile, petiolar node rectangular nodiform; both waist segments strongly sculptured. Abdominal segment III narrowly attaches to abdominal segment IV. Paramere small. Pygostyle absent. Pilosity long, erect to suberect. On forewing, pterostigma well developed. Costal vein (C) present. Media (M) fused with Rs+M. Media (M) never reaching costal margin. Radial sector vein (Rs) never reaching costal margin. Cross-vein 2r-rs connected with radial sector vein posterior to pterostigma. Cross-vein 2rs-m absent. Cross-vein 1m-cu present. Fusion of Rs+M extended distally, so that 1m-cu arises from Rs+M, not from M. Radius vein (R) absent. Cross-vein cu-a proximal to junction between media and cubitus. Cu present. Free section of cubitus present.

#### *Monomorium* Mayr, 1855

Mandible triangular with three or four teeth. Palp formula 5,3. Antennal scrobe absent. Antenna with 13 segments. First funicular segment short, not globular. In full-face view, eye located in front of midlength of head capsule. Ocelli placed well below occipital margin in front view. Occipital carina invisible. With head in full-face view, width excluding eyes is not distinctly narrowed anteriorly from level of posterior margin of eyes: width at level of posterior edge of eyes is not twice as wide as that at level of mandibular insertions. Mesoscutum in profile strongly overhangs pronotum, latter not visible in dorsal view. Notauli absent. With mesopleuron in lateral view, anterodorsal portion lower than highest point of wing process. Protibia with pectinate tibial spur. Mesotibia tibial spur absent. Metatibia tibial spur simple. Aroliae small. Propodeum unarmed and round. Abdominal segment II without peduncle. Abdominal segment III narrowly attaches to abdominal segment IV. Paramere small. Pygostyle present. Pilosity simple throughout body. On forewing, pterostigma well developed. Costal vein (C) absent. Media (M) fused with Rs+M. Media (M) never reaching costal margin. Radial sector vein (Rs) never reaching costal margin. Cross-vein 2r-rs connected with radial sector vein posterior to pterostigma. Cross-vein 2rs-m absent. Cross-vein 1m-cu present. Rs+M absent. Radius vein (R) absent. Cross-vein cu-a proximal to junction between media and cubitus. Cu present. Free section of cubitus present.

#### *Nesomyrmex* Wheeler, 1910

Mandible triangular and distinctly dentate, with five teeth. Palp formula 5,3. Antennal scrobe reduced. Antenna with 13 segments. First funicular segment not globular, shorter than scape. In full-face view, eye located in front of midlength of head capsule Ocelli placed well below occipital margin in front view. Occipital carina sharp but not forming a raised crest. With head in full-face view, width excluding eyes is not distinctly narrowed anteriorly from level of posterior margin of eyes: width at level of posterior edge of eyes is not twice as wide as that at level of mandibular insertions. Mesoscutum in profile strongly overhangs pronotum, latter not visible in dorsal view. Notauli present. With mesopleuron in lateral view, anterodorsal portion lower than highest point of wing process. Protibia with pectinate tibial spur. Mesotibia tibial spur absent. Metatibia tibial spur absent. Aroliae small. Propodeum unarmed. Abdominal segment II with a long anterior peduncle and a low node, spiracle at or behind midlength of peduncle, but in front of level of node. Abdominal segment III narrowly attaches to abdominal segment IV. Paramere large. Pygostyle absent. Sparse pilosity. On forewing, pterostigma well developed. Costal vein (C) present. Media (M) fused with Rs+M. Media (M) never reaching costal margin. Radial sector vein (Rs) reaches costal margin. Cross-vein 2r-rs connected with radial sector vein posterior to pterostigma. Cross-vein 2rs-m absent. Cross-vein 1m-cu absent. Rs+M merge with Rs. Radius vein (R) present. Cross-vein cu-a proximal to junction between media and cubitus. Cu absent. Free section of cubitus absent.

#### *Pheidole* Westwood, 1839

Mandible with 4–7 teeth which decrease in size from apex to base. Palp formula 5,3. Antennal scrobe is absent. Antenna with 13 segments. First funicular segment globular, shorter than scape. In full-face view, eye located in front of midlength of head capsule. Ocelli placed close to occipital margin in front view. Occipital carina invisible. With head in full-face view, width excluding eyes is not distinctly narrowed anteriorly from level of posterior margin of eyes: width at level of posterior edge of eyes is not twice as wide as that at level of mandibular insertions. Mesoscutum in profile strongly overhangs pronotum, latter not visible in dorsal view. Notauli present. With mesopleuron in lateral view, anterodorsal portion lower than highest point of wing process. Protibia with pectinate tibial spur. Mesotibia tibial spur absent. Metatibia tibial spur absent. Aroliae small. Propodeum unarmed. Abdominal segment II with a long anterior peduncle. Abdominal segment III narrowly attaches to abdominal segment IV. Paramere small. Pygostyle present. Sparse pilosity. On forewing, pterostigma well developed. Costal vein (C) present. Media (M) fused with Rs+M. Media (M) never reaching costal margin. Radial sector vein (Rs) never reaching costal margin. Cross-vein 2r-rs connected with radial sector vein posterior to pterostigma. Cross-vein 2rs-m present. Cross-vein 1m-cu present. Fusion of Rs+M extended distally, so that 1m-cu arises from Rs+M, not from M. Radius vein (R) absent. Cross-vein cu-a proximal to junction between media and cubitus. Cu present. Free section of cubitus present.

#### *Pilotrochus* Brown, 1978

Mandible with 4–7 teeth. Palp formula 5,3. Antennal scrobe is reduced. Antenna with 13 segments. First funicular segment globular, shorter than scape. In full-face view, eye located in front of midlength of head capsule. Ocelli placed well below occipital margin in front view. Occipital carina invisible. With head in full-face view, width excluding eyes is distinctly narrowed anteriorly from level of posterior margin of eyes: width at level of posterior margin of eyes is nearly twice as wide as that at level of mandible insertions. Mesoscutum in profile strongly overhangs pronotum, latter not visible in dorsal view. Notauli present. With mesopleuron in lateral view, anterodorsal portion lower than highest point of wing process. Protibia with pectinate tibial spur. Mesotibia tibial spur absent. Metatibia tibial spur absent. Aroliae small. Propodeum unarmed. Abdominal segment II with a long anterior peduncle. Abdominal segment III narrowly attaches to abdominal segment IV. Paramere small. Pygostyle present. Sparse pilosity. On forewing, pterostigma well developed. Costal vein (C) absent. Media (M) fused with Rs+M. Media (M) never reaching costal margin. Radial sector vein (Rs) never reaching costal margin. Cross-vein 2r-rs connected with radial sector vein posterior to pterostigma. Cross-vein 2rs-m absent. Cross-vein 1m-cu present. Fusion of Rs+M extended distally, so that 1m-cu arises from Rs+M, not from M. Radius vein (R) absent. Cross-vein cu-a proximal to junction between media and cubitus. Cu present. Free section of cubitus present.

#### *Pristomyrmex* Mayr, 1866

Mandible edentate. Palp formula 2,2. Antennal scrobe reduced. Antenna with 12 segments. First funicular segment short, not globular, about a third length of second funicular segment. In full-face view, eye located above of base of clypeus. Ocelli placed close to occipital margin in front view. Occipital carina invisible. With head full-face view, width excluding eyes is not distinctly narrowed anteriorly from level of posterior margin of eyes: width at level of posterior edge of eyes is not twice as wide as that at level of mandibular insertions. Mesoscutum in profile strongly overhangs pronotum, latter not visible in dorsal view. Notauli present. With mesopleuron in lateral view, anterodorsal portion lower than highest point of wing process. Protibia with pectinate tibial spur. Mesotibia tibial spur absent. Metatibia tibial spur absent. Aroliae small. Propodeum unarmed. Abdominal segment II with a long anterior peduncle. Abdominal segment III narrowly attaches to abdominal segment IV. Paramere large. Pygostyle present. Pilosity simple throughout body. On forewing, pterostigma well developed. Costal vein (C) absent. Media (M) fused with Rs+M. Media (M) never reaching costal margin. Radial sector vein (Rs) never reaching costal margin. Cross-vein 2r-rs connected with radial sector vein posterior to pterostigma. Cross-vein 2rs-m absent. Cross-vein 1m-cu absent. Rs+M merge with Rs. Radius vein (R) absent. Cross-vein cu-a proximal to junction between media and cubitus. Cu absent. Free section of cubitus absent.

#### *Royidris* Bolton & Fisher, 2014

Mandible triangular and distinctly dentate, with two or three teeth. Palp formula 4,3. Antennal scrobe absent. Antenna with 13 segments. SI 30–52. First funicular segment short and globular. Eyes large, located at or in front of midlength of sides. Ocelli placed close to occipital margin in front view. Occipital carina sharp but not forming a raised crest. With head full-face view, width excluding eyes is not distinctly narrowed anteriorly from level of posterior margin of eyes: width at level of posterior edge of eyes is not twice as wide as that at level of mandibular insertions. Mesoscutum in profile strongly overhangs pronotum, latter not visible in dorsal view. Notauli variably developed, from vestigial to having anterior arms present. With mesopleuron in lateral view, anterodorsal portion lower than highest point of wing process. Protibia with pectinate tibial spur. Mesotibia tibial spur simple. Metatibia tibial spur simple. Aroliae small. Propodeum usually unarmed and rounded, but in some posterodorsal angle is reinforced by a carina, or angle projects as a low, obtuse tooth; propodeal lobes rounded. Abdominal segment II with an anterior peduncle, spiracle at, or slightly in front of, midlength of peduncle, well in front of level of low, rounded node. Abdominal segment II in profile slightly longer than Abdominal segment III. Abdominal segment III narrowly attaches to abdominal segment IV. Paramere large. Pygostyle present. Pilosity simple throughout body. On forewing, pterostigma well developed. Costal vein (C) absent. Media (M) fused with Rs+M. Media (M) never reaching costal margin. Radial sector vein (Rs) never reaching costal margin. Cross-vein 2r-rs connected with radial sector vein posterior to pterostigma. Cross-vein 2rs-m absent. Cross-vein 1m-cu present. Fusion of Rs+M extended distally, so that 1m-cu arises from Rs+M, not from M. Radius vein (R) absent. Cross-vein cu-a proximal to junction between media and cubitus. Cu present. Free section of cubitus absent.

#### *Solenopsis* Westwood, 1840

Mandible with two or three teeth. Palp formula 5,3. Antennal scrobe is reduced. Antenna with 12 segments. First funicular segment globular, shorter than scape. Eyes large, located at or in front of midlength of sides. Ocelli placed near occipital margin in front view. Occipital carina invisible. With head in full-face view, width excluding eyes is not distinctly narrowed anteriorly from level of posterior margin of eyes: width at level of posterior edge of eyes is not twice as wide as that at level of mandibular insertions. Mesoscutum in profile strongly overhangs pronotum, latter not visible in dorsal view. Notauli absent. With mesopleuron in lateral view, anterodorsal portion lower than highest point of wing process. Protibia with pectinate tibial spur. Mesotibia tibial spur absent. Metatibia tibial spur absent. Aroliae small. Propodeum unarmed. Abdominal segment II with a short peduncle. Abdominal segment III narrowly attaches to abdominal segment IV. Paramere small. Pygostyle present. Pilosity simple throughout body. On forewing, pterostigma well developed. Costal vein (C) absent. Media (M) fused with Rs+M. Media (M) never reaching costal margin. Radial sector vein (Rs) never reaching costal margin. Cross-vein 2r-rs connected with radial sector vein posterior to pterostigma. Cross-vein 2rs-m absent. Cross-vein 1m-cu present. Fusion of Rs+M extended distally, so that 1m-cu arises from Rs+M, not from M. Radius vein (R) absent. Cross-vein cu-a proximal to junction between media and cubitus. Cu present. Free section of cubitus present.

#### *Strumigenys* Smith, 1860

Mandible edentate. Palp formula 5,3. Antennal scrobe is absent. Antenna with 13 segments. First funicular segment not subglobular, same size as scape. Eyes large, located at or in front of midlength of sides. Ocelli placed near occipital margin in front view. Occipital carina invisible. With head in full-face view, width excluding eyes is distinctly narrowed anteriorly from level of posterior margin of eyes: width at level of posterior margin of eyes is nearly twice as wide as that at level of mandible insertions. Mesoscutum in profile strongly overhangs pronotum, latter not visible in dorsal view. Notauli absent. With mesopleuron in lateral view, anterodorsal portion lower than highest point of wing process. Protibia with pectinate tibial spur. Mesotibia tibial spur absent. Metatibia tibial spur absent. Aroliae small. Propodeum angle projects as a low, obtuse tooth. Abdominal segment II with a short peduncle. Abdominal segment III narrowly attaches to abdominal segment IV. Paramere small. Pygostyle present. Sparse pilosity. On forewing, pterostigma well developed. Costal vein (C) absent. Media (M) absent. Media (M) absent. Radial sector vein (Rs) never reaching costal margin. Cross-vein 2r-rs connected with radial sector vein posterior to pterostigma. Cross-vein 2rs-m absent. Cross-vein 1m-cu absent. Rs+M absent. Radius vein (R) absent. Cross-vein cu-a absent. Cu absent. Free section of cubitus absent.

#### *Syllophopsis* Santschi, 1915

Mandible with three teeth. Palp formula 5,3. Antennal scrobe reduced. Antenna with 13 segments. First funicular segment short, not globular. Eyes large, located at or in front of midlength of sides. Ocelli placed near occipital margin in front view. Occipital carina invisible. With head in full-face view, width excluding eyes is not distinctly narrowed anteriorly from level of posterior margin of eyes: width at level of posterior edge of eyes is not twice as wide as that at level of mandibular insertions. Mesoscutum in profile strongly overhangs pronotum, latter not visible in dorsal view. Notauli absent. With mesopleuron in lateral view, anterodorsal portion lower than highest point of wing process. Protibia with pectinate tibial spur. Mesotibia tibial spur absent. Metatibia tibial spur absent. Aroliae small. Propodeum unarmed. Abdominal segment II with a short peduncle. Abdominal segment III narrowly attaches to abdominal segment IV. Paramere large. Pygostyle present. Pilosity simple throughout body. On forewing, pterostigma well developed. Costal vein (C) present. Media (M) fused with Rs+M. Media (M) never reaching costal margin. Radial sector vein (Rs) never reaching costal margin. Cross-vein 2r-rs connected with radial sector vein posterior to pterostigma. Cross-vein 2rs-m absent. Cross-vein 1m-cu present. Fusion of Rs+M extended distally, so that 1m-cu arises from Rs+M, not from M. Radius vein (R) absent. Cross-vein cu-a proximal to junction between media and cubitus. Cu present. Free section of cubitus present.

#### *Terataner* Emery, 1912

Mandible triangular and distinctly dentate, with five or six teeth. Palp formula 4,3. Antennal scrobe absent. Antenna with 13 segments. First funicular segment globular, shorter than scape. Eyes large, at or in front of midlength of sides. Ocelli placed near occipital margin in front view. Occipital carina invisible. With head in full-face view, width excluding eyes is not distinctly narrowed anteriorly from level of posterior margin of eyes: width at level of posterior edge of eyes is not twice as wide as that at level of mandibular insertions. Pronotum anterodorsally sharply marginate, with sharp, dentate corners. Notauli absent. With mesopleuron in lateral view, anterodorsal portion is higher than highest point of wing process. Protibia with pectinate tibial spur. Mesotibia tibial spur absent. Metatibia tibial spur simple. Aroliae small. Propodeum unarmed. Abdominal segment II with a long anterior peduncle. Abdominal segment III narrowly attaches to abdominal segment IV. Paramere large. Pygostyle present. Pilosity long, erect to suberect. On forewing, pterostigma well developed. Costal vein (C) absent. Media (M) fused with Rs+M. Media (M) never reaching costal margin. Radial sector vein (Rs) reaches costal margin. Cross-vein 2r-rs connected with radial sector vein posterior to pterostigma. Cross-vein 2rs-m absent. Cross-vein 1m-cu present. Fusion of Rs+M extended distally, so that 1m-cu arises from Rs+M, not from M. Radius vein (R) present. Cross-vein cu-a proximal to junction between media and cubitus. Cu present. Free section of cubitus present.

#### *Tetramorium* Mayr, 1855

Mandible triangular and distinctly dentate, with 4–7 teeth. Palp formula 5,3. Antennal scrobe reduced. Antenna with 10–13 segments. First funicular segment is more distinctly elongated than ors: length is nearly or more than twice as long as third funicular segment. Eyes large, at or in front of midlength of sides. Ocelli placed well below occipital margin in front view. Occipital carina invisible. With head in full-face view, width excluding eyes is not distinctly narrowed anteriorly from level of posterior margin of eyes: width at level of posterior edge of eyes is not twice as wide as that at level of mandibular insertions. Mesoscutum in profile strongly overhangs pronotum, latter not visible in dorsal view. Notauli present. With mesopleuron in lateral view, anterodorsal portion lower than highest point of wing process. Protibia with pectinate tibial spur. Mesotibia tibial spur absent. Metatibia tibial spur simple. Aroliae small. Propodeum armed or angle projects as a low, obtuse tooth. Abdominal segment II with a short peduncle. Abdominal segment III narrowly attaches to Abdominal segment IV. Paramere small. Pygostyle present. Pilosity long, erect to suberect. On forewing, pterostigma well developed. Costal vein (C) absent. Media (M) fused with Rs+M. Media (M) never reaching costal margin. Radial sector vein (Rs) never reaching costal margin. Cross-vein 2r-rs connected with radial sector vein posterior to pterostigma. Cross-vein 2rs-m absent. Cross-vein 1m-cu present. Fusion of Rs+M extended distally, so that 1m-cu arises from Rs+M, not from M. Radius vein (R) absent. Cross-vein cu-a proximal to junction between media and cubitus. Cu present. Free section of cubitus present.

#### *Trichomyrmex* Mayr, 1865

Mandible reduced, short, and narrow, with only two or three teeth. Palp formula 5,3. Antennal scrobe absent. Antenna with 13 segments. First funicular segment subglobular. Eyes large, at or in front of midlength of sides. Ocelli placed well below occipital margin in front view. Occipital carina invisible. With head in full-face view, width excluding eyes is not distinctly narrowed anteriorly from level of posterior margin of eyes: width at level of posterior edge of eyes is not twice as wide as that at level of mandibular insertions. Mesoscutum in profile strongly overhangs pronotum, latter not visible in dorsal view. Notauli absent. With mesopleuron in lateral view, anterodorsal portion lower than highest point of wing process. Protibia with pectinate tibial spur. Mesotibia tibial spur absent. Metatibia tibial spur absent. Aroliae small. Propodeum unarmed. Abdominal segment II with a short peduncle. Abdominal segment III narrowly attaches to abdominal segment IV. Paramere small. Pygostyle absent. Sparse pilosity. On forewing, pterostigma well developed. Costal vein (C) absent. Media (M) fused with Rs+M. Media (M) never reaching costal margin. Radial sector vein (Rs) never reaching costal margin. Cross-vein 2r-rs connected with radial sector vein posterior to pterostigma. Cross-vein 2rs-m absent. Cross-vein 1m-cu absent. Rs+M merge with Rs. Radius vein (R) absent. Cross-vein cu-a proximal to junction between media and cubitus. Cu present. Free section of cubitus absent.

#### *Vitsika* Bolton & Fisher, 2014

Mandible triangular and distinctly dentate, with 5–7 teeth. Palp formula 4,3. Antennal scrobe reduced. Antenna with 13 segments. SI 30–52. First funicular segment short but not globular. Eyes large, located at or in front of midlength of sides. Ocelli placed near occipital margin in front view. Occipital carina sharp but not forming a raised crest. With head in full-face view, width excluding eyes is not distinctly narrowed anteriorly from level of posterior margin of eyes: width at level of posterior edge of eyes is not twice as wide as that at level of mandibular insertions. Mesoscutum in profile strongly overhangs pronotum, latter not visible in dorsal view. Notauli variably developed, from vestigial to having anterior arms present. With mesopleuron in lateral view, anterodorsal portion lower than highest point of wing process. Protibia with pectinate tibial spur. Mesotibia tibial spur absent. Metatibia tibial spur absent. Aroliae small. Propodeum usually unarmed and rounded. Abdominal segment II with an anterior peduncle, spiracle at, or slightly in front of, midlength of peduncle, well in front of level of low, rounded node. Abdominal segment III narrowly attaches to abdominal segment IV. Paramere large. Pygostyle present. Pilosity simple throughout body. On forewing, pterostigma well developed. Costal vein (C) present. Media (M) fused with Rs+M. Media (M) never reaching costal margin. Radial sector vein (Rs) never reaching costal margin. Cross-vein 2r-rs connected with radial sector vein posterior to pterostigma. Cross-vein 2rs-m absent. Cross-vein 1m-cu present. Fusion of Rs+M extended distally so that 1m-cu arises from Rs+M not from M. Radius vein (R) absent. Cross-vein cu-a proximal to junction between media and cubitus. Cu present. Free section of cubitus present.

#### *Vollenhovia* Mayr, 1865

Mandible edentate. Palp formula 2,2. Antennal scrobe absent. Antenna with 13 segments. First funicular equal in size to scape, not globular. Ocelli placed well below occipital margin in front view. Occipital carina invisible. With head in full-face view, width excluding eyes is not distinctly narrowed anteriorly from level of posterior margin of eyes: width at level of posterior edge of eyes is not twice as wide as that at level of mandibular insertions. Mesoscutum in profile strongly overhangs pronotum, latter not visible in dorsal view. Notauli absent with a longitudinal median carina that is narrowly bifurcated anteriorly. With mesopleuron in lateral view, anterodorsal portion lower than highest point of wing process. Protibia with pectinate tibial spur. Mesotibia tibial spur absent. Metatibia tibial spur absent. Aroliae small. Propodeum unarmed. Abdominal segment II without peduncle, in profile petiolar node rectangular nodiform. Abdominal segment III narrowly attaches to abdominal segment IV. Paramere large. Pygostyle absent. Pilosity long, erect to suberect. On forewing, pterostigma reduced in size. Costal vein (C) absent. Media between Rs+M and 2r-rs completely absent. Media (M) absent. Radial sector vein (Rs) never reaching costal margin. Cross-vein 2r-rs present, forming base of “free stigma vein.” Cross-vein 2rs-m absent. Cross-vein 1m-cu absent. Rs+M absent. Radius vein (R) absent. Cross-vein cu-a absent. Cu absent. Free section of cubitus absent.

##### ﻿PONERINAE Lepeletier de Saint-Fargeau, 1835

Diagnosis of male ants of the subfamily Ponerinae in the Malagasy region

- Antenna filiform, consisting of 13 segments.
- Scape not reaching posterior margin of head.
- Mesopleural oblique furrow reaching pronotum far from pronotal posteroventral margin.
- Scuto-scutellar suture usually longitudinally sculptured.
- Abdominal segment II much smaller than segment III in lateral view.
- Abdominal segment II with distinct front, top, and posterior faces in lateral view.
- Abdominal segment II attachment to abdominal segment III narrow and strongly constricted in lateral view.
- Abdominal segment III is nearly as large as abdominal segment IV.
- Cinctus between the segments III and IV distinct and deep.
- Apical portion of abdominal sternum IX not bi-spinose.
- Pygostyles well developed.
- Metatibia with one or two spurs.

Remarks. Our key includes ten Ponerinae genera recorded from the Malagasy region. Overall key modified from Yoshimura and Fisher (2007). Males of *Parvaponera* are unknown were not included in this genera key. *Mesoponera* is known to be paraphyletic (Schmidt and Shattuck 2014). The two species in the Malagasy region, *Mesoponeraambigua* and *Mesoponeramelanariamacra* are keyed out separately.

### ﻿Male-based key to genera of the subfamily Ponerinae

**Table:** 

1	Wings absent	***Hypoponerapunctatissima***
–	Wings present	**2**
2	Mandibles stout and fully developed, masticatory margins overlap completely when mandibles are fully closed (Fig. 60A). Antennal scrobe well defined and extends as long as length of antennal scape	***Platythyrea***
–	Mandibles very reduced in size and lobate, masticatory margins do not overlap completely when mandibles are fully closed (Fig. 60B). Antennal scrobe absent; if weakly defined, then length distinctly shorter than length of antennal scape	**3**
3	Pretarsal claw multidentate to pectinate (Fig. 61A)	***Leptogenys***
–	Pretarsal claw edentate or with at most two preapical teeth (Fig. 61B)	**4**
4	Hind wing with jugal lobe (Fig. 62A)	**5**
–	Hind wing without jugal lobe (Fig. 62B)	**11**
5	Notauli present on mesoscutum (Fig. 63A)	**6**
–	Notauli absent on mesoscutum (Fig. 63B)	**8**
6	Mesometapleural suture deep and sculptured, dorsal margin of abdominal segment II, in frontal view, usually showing two apices (Fig. 64A)	***Anochetusgoodmani***
–	Mesometapleural suture deep but not sculptured, dorsolateral corner of abdominal segment II, in frontal view, not showing two apices (Fig. 64B)	**7**
7	Subpetiolar process in profile view convex ventrally (Fig. 65A). Apical portion of abdominal tergum VIII forming a distinct spine (Fig. 65C)	***Mesoponeramelanariamacra***
–	Subpetiolar process in profile view subtriangular (Fig. 65B). Apical portion of abdominal tergum VIII not forming a spine (Fig. 65D)	***Mesoponeraambigua***
8	Apical portion of abdominal tergum VIII not forming a spine (Fig. 66A)	***Anochetus***
–	Apical portion of abdominal tergum VIII forming a distinct spine (Fig. 66B)	**9**
9	Dorsal margin of abdominal segment II, in frontal view, with single sharp apex (Fig. 67A)	***Odontomachus***
–	Dorsal margin of abdominal segment II, in frontal view, without single sharp apex (Fig. 67B)	**10**
10	In profile view, abdominal segment II surmounted by a thick node (Fig. 68A)	***Bothroponera***
–	In profile view, abdominal segment II node generally scale-like and thin (Fig. 68B)	***Brachyponera*** (Mauritius)
11	Apical portion of abdominal tergum VIII without downcurved spine (Fig. 69A)	***Hypoponera***
–	Apical portion of abdominal tergum VIII with downcurved spine (Fig. 69B)	**12**
12	Ventral apex of meso- and metatibia, when viewed from front with femur at right angle to body, with single spur, spur large and pectinate (Fig. 70A)	***Ponera***
–	Ventral apex of meso- and metatibia, when viewed from front with femur at right angle to body, with two spurs consisting of a larger, pectinate spur and a smaller, simple spur (Fig. 70B)	***Euponera***

**Figure 60.**
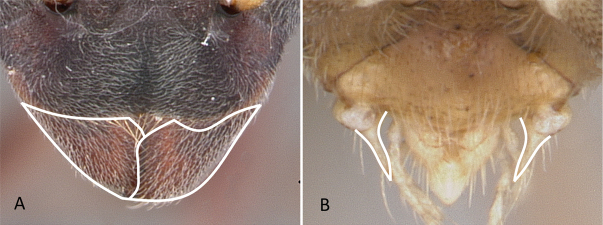
Mandible in full-face view **A***Platythyreaarthuri* (CASENT0442287) **B***Mesoponeraambigua* (CASENT0052325). Photographer April Nobile.

**Figure 61.**
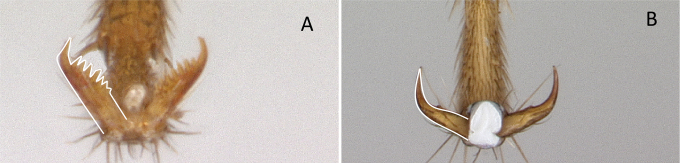
Pretarsal claw **A***Leptogenysmangabe* (CASENT0496777) **B***Bothroponeracambouei* (CASENT0497079). Photographer April Nobile.

**Figure 62.**
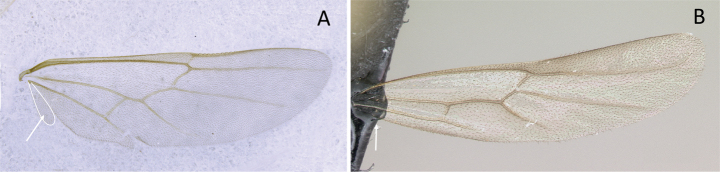
Hind wing **A***Odontomachuscoquereli* (CASENT0740610) **B***Leptogenysmangabe* (CASENT0496777). Photographers Isabella Muratore (**A**) April Nobile (**B**).

**Figure 63.**
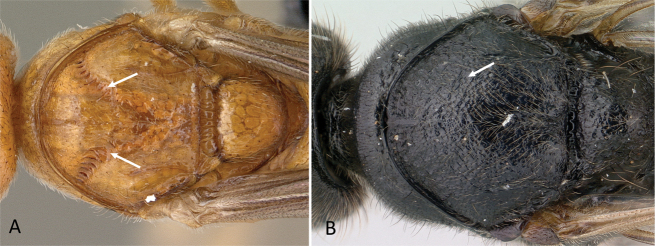
Notauli on mesoscutum **A***Anochetusgoodmani* (CASENT0147683) **B***Bothroponerawasmannii* (CASENT0134532). Photographer Dimby Raharinjanahary.

**Figure 64.**
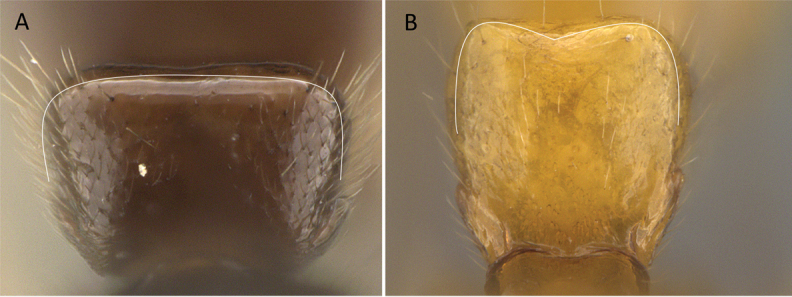
Dorsolateral corner of abdominal segment II in rear view **A***Anochetusgoodmani* (CASENT0147683) **B***Mesoponeraambigua* (CASENT0108325). Photographer Michele Esposito.

**Figure 65.**
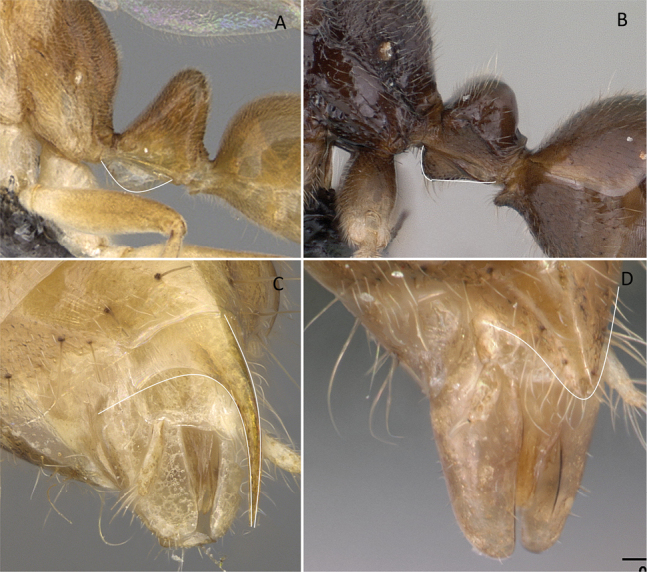
Abdominal segment II in profile view showing the subpetiolar process; apical portion of abdominal tergum VIII **A, C***Mesoponeramelanariamacra* (CASENT0272313) **B, D***Mesoponeraambigua* (CASENT0135592). Photographers Michele Esposito (**A**, **C**), Dimby Raharinjanahary (**B**, **D**).

**Figure 66.**
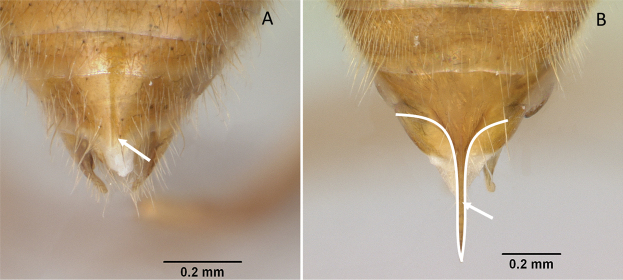
Apical portion of abdominal tergum VIII **A***Anochetusmadagascarensis* (CASENT0442379) **B***Odontomachuscoquereli* (CASENT0049797). Photographer April Nobile.

**Figure 67.**
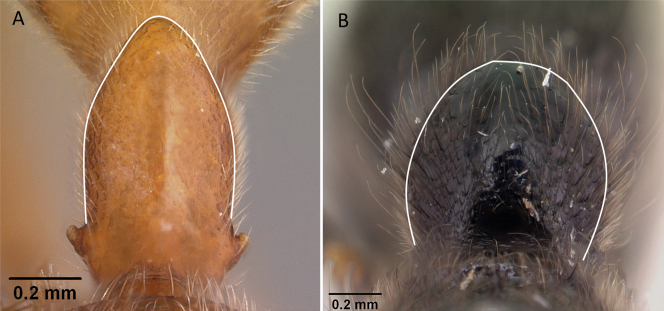
Abdominal segment II in frontal view **A***Odontomachuscoquereli* (CASENT0049797) **B***Bothroponeracambouei* (CASENT0497079). Photographers Masashi Yoshimura (**A**), April Nobile (**B**).

**Figure 68.**
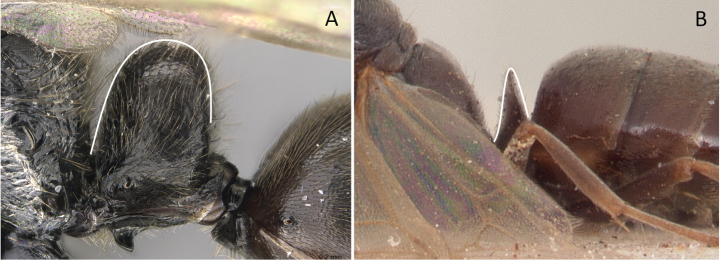
Abdominal segment II form **A***Bothroponerawasmannii* (CASENT0147642) **B***Brachyponerasennaarensis* (https://www.antweb.org/specimen.do?code=SAM-HYM-C002312). Photographer Michele Esposito.

**Figure 69.**
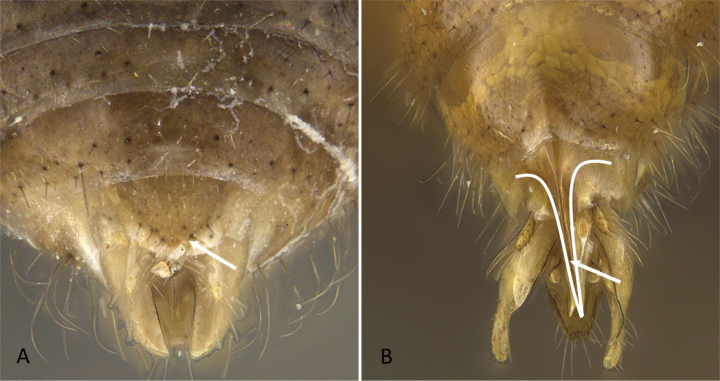
Apical portion of abdominal tergum VIII **A***Hypoponera* mg016 (CASENT0466110) **B***Euponeravohitravo* (CASENT0740617). Photographer Michele Esposito.

**Figure 70.**
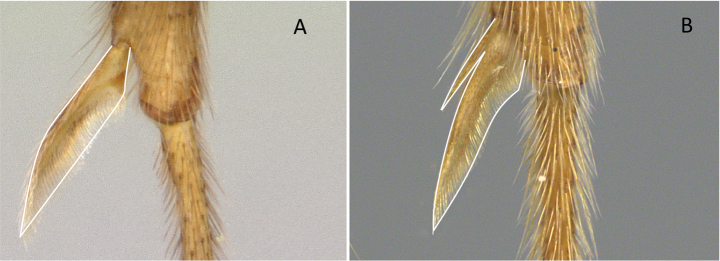
Tibial spur on metatibia **A***Hypoponera* mg057 (CASENT0430684) **B***Euponeravohitravo* (CASENT0740617). Photographers April Nobile (**A**), Michele Esposito (**B**).

#### *Anochetus* Mayr, 1861

All males winged. Antennal scrobe absent. Mandible reduced. Basal cavity of mandible extending to front face, visible in full-face view. Antenna with 13 segments. Notauli absent except for *Anochetusgoodmani*. Mesepimeron with epimeral lobe. In most cases, each dorsolateral corner of abdominal segment II in anterior view with distinct projection. Dorsal margin of abdominal segment II, in anterior view, usually showing two apices. Apical margin of abdominal tergum VIII not projecting into sharp spine. Jugal lobe of hind wing present. Mesotibia and metatibia with two spurs. Claws simple, not multidentate or pectinate. On forewing, pterostigma well developed. Costal vein (C) present. Cross-vein 1m-cu present. Radial sector vein (Rs) complete between M+Rs and 2r-rs. Radial sector vein (Rs) reaches to costal margin. Cross-vein 2r-rs connected with radial sector vein posterior to pterostigma. Cross-vein 2rs-m present. Cross-vein cu-a proximal to junction between media and cubitus. Media between Rs+M and 2rs-m completely present. On hindwing, radius vein (R) absent. Radial sector vein (Rs) present. Cross-vein 1rs-m present. Media (M) usually present. M+Cu present. 1rs-m+M absent. Free section of cubitus present. Cross-vein cu-a present.

The presence of notauli is known for *Anochetus* in the Asian region, including in Vietnam *Anochetusmixtus*, *Anochetusprinceps* and in Indonesia *Anochetusfilicornis*, but only the *goodmani* species exhibits this feature in the Malagasy region.

#### *Bothroponera* Mayr, 1862

Males winged. Antennal scrobe absent. Mandible reduced in size. Basal cavity of mandible extending to front face and visible in full-face view. Antenna with 13 segments. Notauli never impressed on mesoscutum. Mesepimeron with epimeral lobe. Dorsolateral corner of abdominal segment II in anterior view not projecting. Dorsal margin of abdominal segment II, in frontal view, rounded and in profile view, abdominal segment II surmounted by a thick node. Apical margin of abdominal tergum VIII projecting into sharp spine. Jugal lobe of hind wing present. Mesotibia and metatibiae with two spurs. Claws simple, never multidentate or pectinate. On forewing, pterostigma well developed. Costal vein (C) present. Cross-vein 1m-cu present. Radial sector vein (Rs) fully complete between M+Rs and 2r-rs. Radial sector vein (Rs) reaches to costal margin. Cross-vein 2r-rs connected with radial sector vein posterior to pterostigma. Cross-vein 2rs-m present. Cross-vein cu-a proximal to junction between media and cubitus. Media between Rs+M and 2rs-m completely present. On hindwing, radius vein (R) absent. Radial sector vein (Rs) present. Cross-vein 1rs-m present. Media (M) absent. M+Cu present. 1rs-m+M present. Free section of cubitus present. Cross-vein cu-a present.

#### *Brachyponera* Emery, 1900

Males winged. Antennal scrobe absent. Mandible reduced in size. Basal cavity of mandible extending to front face and visible in full-face view. Antenna with 13 segments. Notauli never impressed on mesoscutum. Mesepimeron with epimeral lobe. Dorsolateral corner of abdominal segment II in anterior view not projecting. Dorsal margin of abdominal segment II, in frontal view, rounded and in profile view, petiolar node generally scale-like and thin. Apical margin of abdominal tergum VIII projecting into sharp spine. Jugal lobe of hind wing present. Mesotibia and metatibiae with two spurs. Claws simple, never multidentate or pectinate. On forewing, pterostigma well developed. Costal vein (C) present. Cross-vein 1m-cu present. Radial sector vein (Rs) fully complete between M+Rs and 2r-rs. Radial sector vein (Rs) reaches to costal margin. Cross-vein 2r-rs connected with radial sector vein posterior to pterostigma. Cross-vein 2rs-m present. Cross-vein cu-a located in line to junction between media and cubitus. Media between Rs+M and 2rs-m completely present. On hindwing, radius vein (R) absent. Radial sector vein (Rs) absent. Cross-vein 1rs-m present. Media (M) present. M+Cu present. 1rs-m+M present. Free section of cubitus absent. Cross-vein cu-a present.

#### *Euponera* Forel, 1891.

Males winged. Antennal scrobe absent. Mandible reduced in size. Basal cavity of mandible extending to front face and visible in full-face view. Antenna with 13 segments. Notauli present or absent. Mesepimeron with epimeral lobe. Dorsolateral corner of abdominal segment II in anterior view not projecting. Dorsal margin of abdominal segment II, in frontal view, rounded. Apical margin of abdominal tergum VIII projecting into sharp spine. Jugal lobe of hind wing absent. Mesotibia and metatibiae with two spurs. Claws simple, never multidentate or pectinate. On forewing, pterostigma well developed. Costal vein (C) present. Cross-vein 1m-cu present. Radial sector vein (Rs) fully complete between M+Rs and 2r-rs. Radial sector vein (Rs) reaches to costal margin. Cross-vein 2r-rs connected with radial sector vein posterior to pterostigma. Cross-vein 2rs-m present. Cross-vein cu-a located in line to junction between media and cubitus vein. Media between Rs+M and 2rs-m completely present. On hindwing, radius vein (R) absent. Radial sector vein (Rs) absent. Cross-vein 1rs-m present. Media (M) present. M+Cu present. 1rs-m+M present. Free section of cubitus absent. Cross-vein cu-a present.

#### *Hypoponera* Santschi, 1938

Ergatoid males of Ponerinae are easily distinguished by having: (1) abdominal segment III as large as segment IV; and (2) a distinct constriction between abdominal segments III and IV.

In winged males, antennal scrobe absent. Mandible reduced in size. Basal cavity of mandible extending to front face and visible in full-face view. Antenna with 13 segments. Notauli never impressed on mesoscutum. Mesepimeron without epimeral lobe. Dorsolateral corner of abdominal segment II in anterior view lacking distinct projection. Dorsal margin of abdominal segment II, in anterior view, without a conical or pointed apex. Apical margin of abdominal tergum VIII without spine. Jugal lobe of hind wing absent. Mesotibia and metatibia with single spur. Claws simple, never multidentate or pectinate. On forewing, pterostigma reduced in size. Costal vein (C) present. Cross-vein 1m-cu present. Radial sector vein (Rs) fully complete between M+Rs and 2r-rs. Radial sector vein (Rs) reaches to costal margin. Cross-vein 2r-rs connected with radial sector vein distal to pterostigma. Cross-vein 2rs-m present. Cross-vein cu-a proximal to junction between media and cubitus vein. Media between Rs+M and 2rs-m completely present. On hindwing, radius vein (R) absent. Radial sector vein (Rs) absent. Cross-vein 1rs-m present. Media (M) present. M+Cu present. 1rs-m+M present. Free section of cubitus absent. Cross-vein cu-a present.

#### *Leptogenys* Roger, 1861

Males winged. Antennal scrobe absent. Mandible reduced in size. Basal cavity of mandible extending to front face and visible in full-face view. Antenna with 13 segments. Notauli impressed on mesoscutum in most species. Mesepimeron with epimeral lobe. Dorsolateral corner of abdominal segment II in anterior view without distinct projections. Dorsal margin of abdominal segment II in anterior view gently rounded, not forming a conical or pointed apex. Apical margin of abdominal tergum VIII occasionally featuring downcurved projection. Jugal lobe of hindwing absent in most species. Mesotibia and metatibia with two spurs. Pretarsal claw multidentate to pectinate. On forewing, pterostigma well developed. Costal vein (C) present. Cross-vein 1m-cu present. Radial sector vein (Rs) fully complete between M+Rs and 2r-rs. Radial sector vein (Rs) reaches to costal margin. Cross-vein 2r-rs connected with radial sector vein posterior to pterostigma. Cross-vein 2rs-m present. Cross-vein cu-a proximal to junction between media and cubitus. Media between Rs+M and 2rs-m completely present. On hindwing, radius vein (R) absent. Radial sector vein (Rs) absent. Cross-vein 1rs-m present. Media (M) present. M+Cu present. 1rs-m+M present. Free section of cubitus absent. Cross-vein cu-a present.

#### *Mesoponera* Emery, 1900

*Mesoponeraambigua* André, 1890. Males winged. Antennal scrobe absent. Mandible reduced in size. Basal cavity of mandible extending to front face and visible in full-face view. Antenna with 13 segments. Notauli impressed on mesoscutum. Mesepimeron with epimeral lobe. Dorsolateral corner of abdominal segment II in anterior view not projecting. Dorsal margin of abdominal segment II, in frontal view, rounded. Subpetiolar process in profile view subtriangular. Apical portion of abdominal tergum VIII without downcurved spine. Jugal lobe of hind wing present. Mesotibia and metatibiae with two spurs. Claws simple, never multidentate or pectinate. On forewing, pterostigma well developed. Costal vein (C) present. Cross-vein 1m-cu present. Radial sector vein (Rs) fully complete between M+Rs and 2r-rs. Radial sector vein (Rs) reaches to costal margin. Cross-vein 2r-rs connected with radial sector vein posterior to pterostigma. Cross-vein 2rs-m present. Cross-vein cu-a located in line to junction between media and cubitus. Media between Rs+M and 2rs-m completely present. On hindwing, radius vein (R) absent. Radial sector vein (Rs) present. Cross-vein 1rs-m present. Media (M) present. M+Cu present. Free section of cubitus present. Cross-vein cu-a present.

*Mesoponeramelanariamacra* Emery, 1894. Males winged. Antennal scrobe absent. Mandible reduced in size. Basal cavity of mandible extending to front face and visible in full-face view. Antenna with 13 segments. Notauli impressed on mesoscutum. Mesepimeron with epimeral lobe. Dorsolateral corner of abdominal segment II in anterior view not projecting. Dorsal margin of abdominal segment II, in frontal view, rounded. Subpetiolar process in profile view convex ventrally. Apical portion of abdominal tergum VIII with downcurved spine. Jugal lobe of hind wing present. Mesotibia and metatibiae with two spurs. Claws simple, never multidentate or pectinate. On forewing, pterostigma well developed. Costal vein (C) present. Cross-vein 1m-cu present. Radial sector vein (Rs) fully complete between M+Rs and 2r-rs. Radial sector vein (Rs) reaches to costal margin. Cross-vein 2r-rs connected with radial sector vein posterior to pterostigma. Cross-vein 2rs-m present. Cross-vein cu-a proximal to junction between media and cubitus. Media between Rs+M and 2rs-m completely present. On hindwing, radius vein (R) present. Radial sector vein (Rs) present. Cross-vein 1rs-m present. Media (M) present. M+Cu present. Free section of cubitus present. Cross-vein cu-a present.

#### *Odontomachus* Latreille, 1804

Males winged. Antennal scrobe absent. Mandible reduced. Basal cavity of mandible extending to front face and visible in full-face view. Antenna with 13 segments. Notauli never impressed on mesoscutum. Mesepimeron with epimeral lobe. Dorsolateral corner of abdominal segment II in anterior view not projecting. Dorsal margin of abdominal segment II in anterior view more or less conical, with a narrowly rounded or pointed apex. Apical margin of abdominal tergum VIII projecting into a sharp spine. Jugal lobe of hind wing present. Mesotibia and metatibia with two spurs. Claws simple, never multidentate to pectinate. On forewing, pterostigma well developed. Costal vein (C) present. Cross-vein 1m-cu present. Radial sector vein (Rs) fully complete between M+Rs and 2r-rs. Radial sector vein (Rs) reaches to costal margin. Cross-vein 2r-rs connected with radial sector vein posterior to pterostigma. Cross-vein 2rs-m present. Cross-vein cu-a proximal to junction between media and cubitus. Media between Rs+M and 2rs-m completely present. On hindwing, radius vein (R) absent. Radial sector vein (Rs) absent. Cross-vein 1rs-m present. Media (M) present. M+Cu present. 1rs-m+M present. Free section of cubitus absent. Cross-vein cu-a present.

#### *Parvaponera* Schmidt & Shattuck, 2014

While the male of this species remains unknown worldwide, the analysis of wing venation and morphological characteristics based on the gyne might be helpful to identify the male of this species in the future.

**Figure 71.**
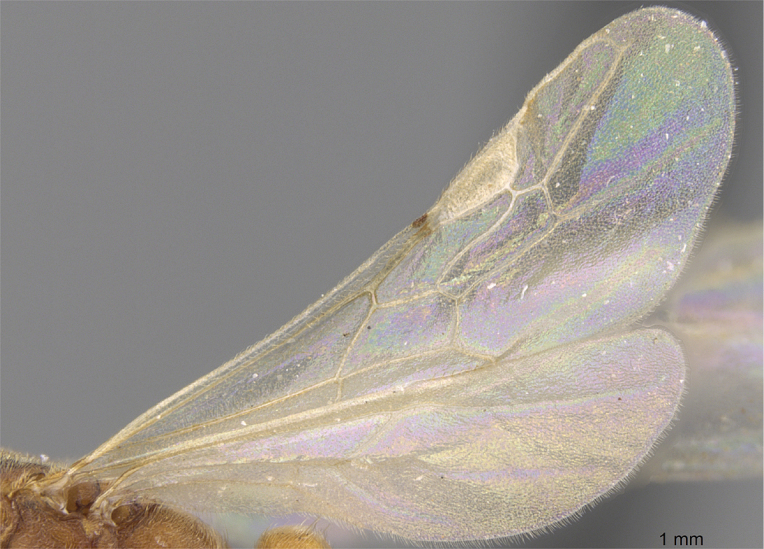
Forewing venation in queen caste. *Parvaponeradarwiniimadecassa* (CASENT0410199). Photographer Cerise Chen.

Queen: Antenna with 12 segments. Mesotibia and metatibia with two spurs. Claws simple, never multidentate to pectinate. On forewing (Fig. 71), pterostigma well developed. Costal vein (C) present. Cross-vein 1m-cu present. Radial sector vein (Rs) fully complete between M+Rs and 2r-rs. Radial sector vein (Rs) reaches to costal margin. Cross-vein 2r-rs connected with radial sector vein posterior to pterostigma. Cross-vein 2rs-m present. Cross-vein cu-a distal to junction between media and cubitus. Media between Rs+M and 2rs-m completely present.

#### *Platythyrea* Roger, 1863

Males winged. Antennal scrobe distinct. Mandible large, stout, triangular, with many teeth on masticatory margin, and masticatory margins completely overlap when mandibles are fully closed. Basal cavity of mandible invisible in full-face view. Antenna with 13 segments. Notauli impressed on mesoscutum. Mesepimeron with epimeral lobe. Dorsolateral corner of abdominal segment II in anterior view lacking distinct projection. Dorsal margin of abdominal segment II, in anterior view, broadly or narrowly rounded. Apical margin of abdominal tergum VIII does not project strongly into sharp spine. Jugal lobe of hind wing may or may not be present. Mesotibia and metatibiae with two spurs. Claws simple, never multidentate or pectinate. Body surface sparsely punctate. On forewing, pterostigma well developed. Costal vein (C) present. Cross-vein 1m-cu present. Radial sector vein (Rs) fully complete between M+Rs and 2r-rs. Radial sector vein (Rs) reaches to costal margin. Cross-vein 2r-rs connected with radial sector vein posterior to pterostigma. Cross-vein 2rs-m present. Cross-vein cu-a located in line to junction between media and cubitus. Media between Rs+M and 2rs-m completely present. On hindwing, radius vein (R) absent. Radial sector vein (Rs) absent. Cross-vein 1rs-m present. Media (M) present. M+Cu present. 1rs-m+M present. Free section of cubitus absent. Cross-vein cu-a present.

#### *Ponera* Latreille, 1804

Males winged. Antennal scrobe absent. Mandible reduced in size. Basal cavity of mandible extending to front face, visible in full-face view. Antenna with 13 segments. Notauli never impressed on mesoscutum. Mesepimeron without epimeral lobe. Dorsolateral corner of abdominal segment II in anterior view lacking distinct projection. Dorsal margin abdominal segment II, in anterior view, without narrowly rounded or pointed apex. Apical margin of abdominal tergum VIII strongly projecting into a sharp spine. Jugal lobe of hind wing absent. Mesotibia and metatibiae with single spur. Claws simple, never multidentate or pectinate. On forewing, pterostigma well developed. Costal vein (C) present. Cross-vein 1m-cu present. Radial sector vein (Rs) fully complete between M+Rs and 2r-rs. Radial sector vein (Rs) reaches to costal margin. Cross-vein 2r-rs connected with radial sector vein posterior to pterostigma. Cross-vein 2rs-m present. Cross-vein cu-a proximal to junction between media and cubitus. Media between Rs+M and 2rs-m completely present. On hindwing, radius vein (R) absent. Radial sector vein (Rs) absent. Cross-vein 1rs-m present. Media (M) present. M+Cu present. 1rs-m+M present. Free section of cubitus absent. Cross-vein cu-a present.

##### ﻿PROCERATIINAE Emery, 1895

Diagnosis of male ants of the subfamily Proceratiinae in the Malagasy region

- Antenna filiform, consisting of 13 segments.
- Scape not reaching posterior margin of head.
- Mesopleural oblique furrow reaching pronotum close to pronotal posteroventral margin.
- Scuto-scutellar suture usually longitudinally sculptured.
- Abdominal segment II attached to abdominal segment III ventrally.
- Abdominal segment II much smaller than segment III in lateral view.
- Abdominal segment II broadly and dorsally attached to abdominal segment III.
- Apical portion of abdominal sternum IX not bi-spinose.
- Pygostyles absent or present.
- Metatibia with one spur.

Remarks. Our key includes three Proceratiinae genera recorded from the Malagasy region. Key modified from Yoshimura and Fisher (2009).

### ﻿Male−based key to genera of the subfamily Proceratiinae

**Table:** 

1	Frontal carinae diverging posteriorly or subparallel, but never merged into single carina (Fig. 72A). Cubitus (Cu) in hindwing present, rarely reduced but with short branch	***Proceratium***
–	Frontal carinae merged into single median carina between antennal sockets (Fig. 72B). Cubitus (Cu) in hindwing absent	**2**
2	Stigmal vein absent: Radial sector vein (Rs) fully present in forewing, joining Radius vein (R) at apical costal margin (Fig. 73A). Pygostyles present	***Discothyrea***
–	Stigmal vein present: Radial sector vein (Rs) absent in medial section of forewing and not reaching costal margin; Radius vein (R) absent on costal margin (Fig. 73B). Pygostyles absent	***Probolomyrmex***

**Figure 72.**
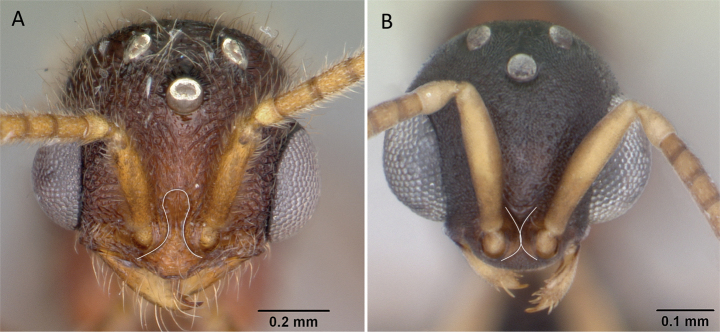
Head in full-face view showing the frontal carinae **A***Proceratium* mgm09 (CASENT0081854) **B***Probolomyrmexcurculiformis* (CASENT0080551). Photographer April Nobile.

**Figure 73.**
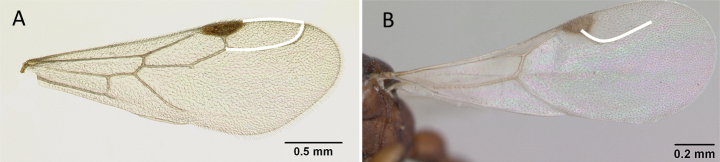
Forewing venation **A***Discothyrea* mgm01 (CASENT0083649) **B***Probolomyrmexcurculiformis* (CASENT0050214). Photographers Erin Prado (**A**), April Nobile (**B**).

#### *Discothyrea* Roger, 1863

Mandible smaller than in conspecific worker, but also triangular to subtriangular. Frontoclypeal region projecting dorsally. Frontal carinae merged into a single median carina. Antennal sockets opening posteriorly. Antenna with 12–13 segments. Labrum bilobed apically. Second segment of maxillary palp not hammer-shaped. Pro-, meso-, and metatibia with a single spur. Pygostyles present. On forewing, pterostigma well developed. Costal vein (C) present. Cross-vein 1m-cu absent. Radial sector vein (Rs) fused to M+Rs. Radial sector vein (Rs) reaches to costal margin. Cross-vein 2r-rs connected with radial sector vein posterior to pterostigma. Cross-vein 2rs-m absent. Cross-vein cu-a proximal to junction between media and cubitus vein. Media between Rs+M and 2rs-m completely absent. On hindwing, radius vein (R) absent. Radial sector vein (Rs) present. Cross-vein 1rs-m absent. Media (M) absent. M+Cu absent. 1rs-m+M absent. Free section of cubitus absent. Cross-vein cu-a absent.

#### *Probolomyrmex* Mayr, 1901

Mandible smaller than in conspecific worker, but also triangular to subtriangular. Frontoclypeal region projecting dorsally. Frontal carinae merged into single median carina. Antennal socket opening posteriorly. Antenna with 13 segments. Labrum bilobed apically. Second segment of maxillary palp hammer-shaped. Pro-, meso-, and metatibia with a single spur. Pygostyles absent. On forewing, pterostigma well developed. Costal vein (C) present. Cross-vein 1m-cu absent. Radial sector vein (Rs) absent between M+Rs and 2r-rs. Radial sector vein (Rs) fails to reach costal margin. Cross-vein 2r-rs present, forming base of “free stigma vein.” Cross-vein 2rs-m absent. Cross-vein cu-a proximal to junction between media and cubitus vein. Media between Rs+M and 2rs-m completely absent. On hindwing, radius vein (R) absent. Radial sector vein (Rs) present. Cross-vein 1rs-m absent. Media (M) absent. M+Cu absent. 1rs-m+M absent. Free section of cubitus absent. Cross-vein cu-a absent.

#### *Proceratium* Roger, 1863

Mandible smaller than in conspecific worker, but also triangular to subtriangular. Frontoclypeal region not projecting dorsally. Frontal carinae separated, not merged into single median carina. Antennal sockets opening dorsally. Antenna with 13 segments. Labrum bilobed apically. Second segment of maxillary palp hammer-shaped. Pro-, meso-, and metatibia with a single spur. Pygostyles absent. On forewing, pterostigma well developed. Costal vein (C) present. Cross-vein 1m-cu absent. Radial sector vein (Rs) absent between M+Rs and 2r-rs. Radial sector vein (Rs) fails to reach costal margin. Cross-vein 2r-rs connected with radial sector vein posterior to pterostigma. Cross-vein 2rs-m absent. Cross-vein cu-a proximal to junction between media and cubitus vein. Media between Rs+M and 2rs-m completely present. On hindwing, radius vein (R) absent. Radial sector vein (Rs) present. Cross-vein 1rs-m present. Media (M) usually present. M+Cu present. 1rs-m+M present. Free section of cubitus present. Cross-vein cu-a present.

##### ﻿PSEUDOMYRMICINAE Smith, 1952

Diagnosis of male *Tetraponera* in the subfamily Pseudomyrmicinae in the Malagasy region.

- Antenna filiform, consisting of 12 segments.
- Abdominal segment II nearly as large as segment III in lateral view.
- Mesopleural oblique furrow reaching pronotum far from pronotal posteroventral margin.
- Apical portion of abdominal sternum IX not bi-spinose.
- Pygostyles present.
- Protibia with one spur.
- Mesotibia with two spurs.
- Metatibia with two spurs.

Mandible triangular and distinctly dentate. Masticatory margin with 2–6 teeth. Anterior margin of clypeus straight to broadly convex, rarely emarginate. Palp formula 6,4. Antennal scrobe absent. Antenna with 12 segments. First funicular segment short and globular. Eyes large, located at or in front of midlength of sides. Ocelli conspicuous. Occipital carina sharp but not forming a raised crest. Promesonotal suture visible in profile or dorsally. Notauli absent. Protibia with pectinate tibial spur. Meso- and metatibiae with two tibial spurs. Aroliae small. Propodeum usually unarmed and rounded. Propodeal spiracle rounded. Abdominal segment III narrowly attaches to abdominal segment IV. Paramere large. Pygostyle present. On forewing, pterostigma well developed but not pigmented. Costal vein (C) absent. Media (M) fused with Rs+M. Media (M) never reaching costal margin. Radial sector vein (Rs) reaches costal margin. Cross-vein 2r-rs connected with radial sector vein posterior to pterostigma. Cross-vein 2rs-m absent. Cross-vein 1m-cu present. Fusion of Rs+M extended distally, so that 1m-cu arises from Rs+M, not from M. R present. Cu-a proximal to junction between media and cubitus vein. Cu present. Free section of cubitus present. On hindwing, radius vein (R) absent. Radial sector vein (Rs) present. Cross-vein 1rs-m present. Media (M) present. M+Cu absent. 1rs-m+M present. Free section of cubitus absent. Cross-vein cu-a absent.

## ﻿Additional information

### Author ORCIDs

Manoa M. Ramamonjisoa https://orcid.org/0009-0005-7805-4433

Nicole Rasoamanana https://orcid.org/0000-0001-9702-7231

Brian L. Fisher https://orcid.org/0000-0002-4653-3270

### Data availability

All of the data that support the findings of this study are available in the main text.
